# Associations of self-reported occupational exposures and settings to ALS: a case–control study

**DOI:** 10.1007/s00420-022-01874-4

**Published:** 2022-05-20

**Authors:** Stephen A. Goutman, Jonathan Boss, Christopher Godwin, Bhramar Mukherjee, Eva L. Feldman, Stuart A. Batterman

**Affiliations:** 1 Department of Neurology, University of Michigan, 1500 E Medical Center Dr, Ann Arbor, MI 48109-5223 USA; 2 NeuroNetwork for Emerging Therapies, University of Michigan, Ann Arbor, MI USA; 3 Department of Biostatistics, University of Michigan, Ann Arbor, MI USA; 4 Department of Environmental Health Sciences, University of Michigan, Ann Arbor, MI USA

**Keywords:** Amyotrophic lateral sclerosis, Occupation, Risk factors, Exposome, Metals

## Abstract

**Background:**

Environmental exposures contribute to the pathogenesis of amyotrophic lateral sclerosis (ALS), a fatal and progressive neurological disease. Identification of these exposures is important for targeted screening and risk factor modification.

**Objective:**

To identify occupational exposures that are associated with a higher risk of ALS using both survey and standard occupational classification (SOC) coding procedures, and to highlight how exposure surveys can complement SOC coding.

**Methods:**

ALS participants and neurologically healthy controls recruited in Michigan completed a detailed exposure assessment on their four most recent and longest held occupations. Exposure scores were generated from the exposure survey, and occupations were assigned to SOC codes by experienced exposure scientists.

**Results:**

This study included 381 ALS and 272 control participants. ALS participants reported higher duration-adjusted occupational exposure to particulate matter (OR = 1.45, 95% CI 1.19–1.78, *p* < 0.001), volatile organic compounds (OR = 1.22, 95% CI 1.02–1.45, *p* = 0.029), metals (OR = 1.48, 95% CI 1.21–1.82, *p* < 0.001), and combustion and diesel exhaust pollutants (OR = 1.20, 95% CI 1.01–1.43, *p* = 0.041) prior to ALS diagnosis, when adjusted for sex, age, and military service compared to controls. In multivariable models, only occupational exposure to metals remained significant risk (OR = 1.56, 95% CI 1.11–2.20, *p* = 0.011), although in an adaptive elastic net model, particulate matter (OR = 1.203), pesticides (OR = 1.015), and metals (1.334) were all selected as risk factors. Work in SOC code “Production Occupations” was associated with a higher ALS risk. SOC codes “Building and Grounds Cleaning and Maintenance Occupations”, “Construction and Extraction Occupations”, “Installation, Maintenance, and Repair Occupations”, and “Production Occupations” were all associated with a higher exposure to metals as determined using survey data.

**Discussion:**

Occupational exposure to particulate matter, volatile organic compounds, metals, pesticides, and combustion and diesel exhaust and employment in “Production Occupations” was associated with an increased ALS risk in this cohort.

**Supplementary Information:**

The online version contains supplementary material available at 10.1007/s00420-022-01874-4.

## Introduction

Amyotrophic lateral sclerosis (ALS) is a fatal neurodegenerative disease that results in degeneration of the motor neuron cells located in the brain, brainstem, and spinal cord causing painless progressive weakness involving cranial and limb muscles along with respiratory failure (Goutman 2017). This relentless progression leads to death within 2–4 years from symptom onset for most individuals that develop this disease. In addition to the motor involvement, up to half of patients with ALS will manifest cognitive changes. The major pathologic hallmark of ALS is aggregation of transactive response (TAR) DNA-binding protein 43 (TDP-43) in multiple brain areas. Approximately 85% of ALS is considered sporadic with no one single mutation underlying the disorder. Among the remaining 15% of familial cases, the most common genetic form is secondary to a hexanucleotide expansion in chromosome 9 open reading frame 72 (*C9orf72*). While the full picture of what causes ALS is incomplete, a combination of genetic and environmental factors is strongly implicated as underlying disease risk and progression (Goutman 2017).

The fact that ALS exhibits incomplete heritability and follows a multistep model of disease adds credence to the hypothesis that environmental exposures contribute to disease (Al-Chalabi and Hardiman 2013; Al-Chalabi et al. 2014). A recent meta-analysis identified exposures to lead, heavy metals, pesticides, agricultural chemicals, solvents, and electric shock as strong ALS risk factors (Wang et al. 2017). Other risk factors are also linked to ALS including smoking, military service, and physical activity (Al-Chalabi and Hardiman 2013). Our group has shown that self-reported residential pesticide exposure and concentrations of persistent organic pollutants in blood are associated with a higher odds of having ALS (Su et al. 2016; Yu et al. 2014). Further, we have shown that higher concentrations of these persistent organic pollutants in blood are associated with a faster disease progression. (Goutman et al. 2019)

Identifying the specific exposures that contribute to ALS risk is a critical step toward better understanding disease pathogenesis and developing mechanism-based therapies. This knowledge will point to specific exposures that should be avoided to decrease ALS risk and prevent disease (Goutman and Feldman 2020). The occupational setting is an important exposure environment, and occupational exposures to metals including lead, pesticides, silica, asbestos, organic dust, contact with animals or fresh animal products, endotoxins, polycyclic aromatic hydrocarbons, and diesel motor exhaust have all been associated with an increased ALS risk (Visser et al. 2019; Malek et al. 2014; Dickerson et al. 2019). Specific occupational sectors associated with an increased ALS risk include mechanics, manufacturing, mechanical, military, painting, precision metal, and/or construction industries (Andrew et al. 2020, 2017; Fang et al. 2009). Additional investigations are needed to elucidate which occupations have a high ALS risk and the job- and task-specific exposures that increase this risk.

The overall goal of this work is to identify occupational exposures that contribute to the risk of developing ALS. We also show how survey data can be used to develop occupational exposure scores for use in ALS disease risk models and demonstrate how these scores complement traditional job codes by providing greater specificity and personal-level details that may affect exposure and risks.

## Methods

### Participants

All patients with an El Escorial diagnosis of ALS seen at the University of Michigan (UM) Pranger ALS Clinic were asked to participate as ALS participants. Controls were identified for this study using an online recruitment database hosted by the Michigan Institute for Clinical & Health Research, which allows University of Michigan research teams to contact individuals that express interest in research participation. Interested controls were selected if they met inclusion criteria and fit the demographic ranges of ALS participants. Controls were excluded if they had a neurodegenerative condition or had a first- or second-degree blood relative with ALS. All participants were older than 18 years and provided verbal and written consent in English, and controls received $50 compensation for study participation and donated blood and urine samples at enrollment. The study received Institutional Review Board approval (HUM28826). Details of this study are previously published. (Su et al. 2016; Yu et al. 2014; Goutman et al. 2019).

### Survey administration and follow-up

Following consent, participants were provided a written questionnaire and completion instructions. In the event a questionnaire was not returned, follow-up phone calls were placed to the participants to encourage completion; for ALS participants, survey completion was also encouraged at follow-up clinic visits. In circumstances where a response was incomplete or illegible, follow-up phone calls were placed to the participants. In some deceased ALS participants or those with severe dysarthria, next of kin were able to provide clarification to responses.

### Survey description and exposure scores derivation

The survey was constructed from instruments available from the Agency for Toxic Substances and Disease Registry (ATSDR) (ATSDR 2000) and input from experts trained in exposure science. The questionnaire queried exposures at four jobs: the most recent; the job before the most recent; and the next two longest held jobs. For ALS participants, jobs that began after onset of symptoms were excluded, as were jobs for ALS participants without an onset date. Each respondent was also asked to provide a complete job history including job title, description, and years worked.

The questionnaire data provided insight into the 9 *exposure types*: particulate matter (PM), volatile organic compounds (VOCs), pesticides, metals, biologicals, combustion/diesel exhaust, electromagnetic radiation, radiation, and corrosives (Table S1). Within each exposure type, we developed multiple *exposure factors* to assess the potential for exposure from specific sources or activities. For example, PM exposure may be contributed by occupational tasks, such as welding, handling dust-generating materials (e.g., talc, powders, fibers), and being exposed to diesel exhaust. Further, chronic exposure will be increased with jobs held for long periods.

The exposure factors were quantified using the survey data, typically using responses to combination of questions (Table S1). For example, occupational PM exposure utilized two factors (likelihood of general PM exposure, and exposures to specific PM sources), and a total of 18 survey questions. This analysis was repeated for each qualifying job reported by each participant. An *occupational exposure score* was obtained for each exposure type and job by summing the exposure factor scores weighted by our assessment of the contribution of the factor to the overall exposure; these were subsequently normalized (0 indicated no exposure potential; 1 indicated the highest possible exposure potential) to facilitate comparisons among exposure types. Finally, the *duration-adjusted occupational exposure score* used the occupational exposure score multiplied by the duration of the job, based on the individual’s job history (job start and end dates). Thus, the *occupational exposure score* represents whether the participant was ever exposed on a particular job, whereas the duration-adjusted occupational *exposure score* accounts for the duration of exposure on up to four jobs.

### Assignment and review of job codes

Job titles and descriptions for all occupations (*N* = 2169, prior to removing excluded jobs, such as those occurring after symptom onset or consent) were processed using two automated job coding platforms: Standardized Occupation Coding for Computer-assisted Epidemiological Research (SOCcer (Russ et al. 2016), available via National Cancer Institute, https://soccer.nci.nih.gov/soccer/); and The National Institute for Occupational Safety and Health (NIOSH) Industry and Occupation Computerized Coding System (NIOCCS; available from https://www.cdc.gov/niosh/topics/coding/code.html). For SOCcer, model version 2.0 was selected and the input file included job titles and job tasks. The system returned 10 SOC codes per job and the fit score for each. For NIOCCS, the 2010 coding scheme was selected, and the input file included Industry Title, Occupation Title, and Job Duties. Both packages were accessed in April 2020. Because both platforms provided the SOC (Standard Occupational Classification), we elected to use SOC as the coding mechanism. The SOCcer and NIOCCS output files were then merged, resulting in multiple SOCs for each of the participants’ jobs. Because SOCs assigned by automated platforms can involve considerable uncertainty, particularly for exposure assessment purposes, we subjected results to a series of validation steps, as discussed below. These steps were performed blinded to ALS or control participant status.

The assigned job codes/titles were first prioritized for manual review using a priority score of 0, 1, 2 or 3, representing “indeterminate,” “low,” “medium,” or “high” priority for review, respectively. Indeterminate was assigned (22 jobs) if the self-described job title and description were judged insufficient to utilize the SOCcer or NIOCCS procedure (although these still provided a result in many circumstances), e.g., titles of “CEO,” “owner,” “division controller,” “management,” or “engineering equipment officer” without additional job descriptors were judged insufficient to allow classification. Low priority was assigned if SOC codes were consistent with high fit, or if there was little potential for significant occupational exposure based on the job title, job description and SOC codes. Second, participants with job titles that suggested low priority but with descriptors that provided supporting information received an additional point (no score exceeded 3), e.g., a job title of secretary (ordinarily receiving a score of 1) for an individual who worked in a military facility was assigned a score of 2. This review was facilitated by sorting by the initially assigned SOCcer codes. Third, we identified jobs for manual review by considering those jobs with priority scores of 2 or 3 if the SOCcer and NIOCCS algorithm outcomes did not agree (*n* = 382), and all jobs with priority scores of 2 or 3 if the SOC fit score was low (< 0.3; *n* = 298). For these jobs, we examined the SOC assignment, and the respondent-provided job titles and descriptors. In some circumstances, we overrode the SOC assignment, drawing first from the top 100 SOC codes assigned, but utilizing additional SOC codes if none of the top 100 were appropriate.

To maintain independence from the survey-derived exposure scores detailed in the previous section, the validation steps did not use survey information. An exposure scientist (C.G.) provided both the initial prioritization and the manual review, which was then checked by a second exposure scientist (S.A.B.), and in a few circumstances, the assignment was revised. After this review, we estimated the misclassification rate using a randomly selected subset (excluding participant jobs that were manually revised and indeterminate jobs). In this subset of jobs (*n* = 117), 8.6% (*n* = 10) were judged to be incorrect. However, all of these jobs occurred in the low priority set. While our analysis is limited in sample size and other regards, it suggests that while the automated coding procedures have a non-negligible misclassification rate, most mistakes occur in occupations with relatively little potential for occupational exposure.

### Data management

All survey data were entered into Redcap by research staff. A number of quality assurance techniques were employed: (1) Redcap logic and data validation tools were utilized; (2) in a random sample of surveys, data were double entered and reviewed; and (3) audits of data were performed to ensure logical responses. Participant were contacted if appropriate for any follow-up questions.

### Statistical analysis

Demographic characteristics of study participants were collected and responses for occupational exposure scores were tabulated by ALS and control status and by demographic characteristics (age, sex, education) for all participants. Continuous data were summarized by mean, standard deviation, median, and interquartile range, and categorical data by counts and percentages. Differences between groups were determined via *t*-test for continuous variables and chi-square tests for categorical variables. For eight subjects with missing military service, single imputation with the mode was used.

### Occupational exposure score analysis

Descriptive statistics and missingness for each occupational exposure score were summarized by ALS-control status. Differences between ALS participants and controls were evaluated using permutation tests. Occupational exposure scores were regressed one-at-a-time against ALS/control status adjusted for age (quartiles), sex, and military service to identify associations between exposure and ALS risk. Nonlinearity in the one-at-time associations between exposure scores and ALS/control status was assessed using generalized additive models adjusted for age (quartiles), sex, and military service. We also considered both an unpenalized and adaptive elastic net penalized multivariable logistic regression model including all nine occupational exposure scores adjusted for age (quartiles), sex, and military service to account for moderately high correlations among several exposures. All analyses were performed for both the occupational exposure score and duration-adjusted occupational exposure scores. We present the duration-adjusted occupational exposure scores in the main text given that they account for the duration of exposure and provide the non-duration-adjusted scores in the supplement.

### SOC code analysis

The number of unique ALS participants and controls, and the corresponding job-years worked, was tabulated within each two-digit SOC code. One sample test of proportions was performed for an enrichment of ALS participants relative to the overall distribution of ALS and control participants in the study population. For each two-digit SOC code, unadjusted and adjusted logistic regression models were used to associate job-years worked with ALS/control status. We then fitted an adjusted logistic regression model with adaptive lasso penalization including all two-digit SOC codes simultaneously to select the two-digit SOC codes associated with ALS risk. From there, job-years corresponding to the selected two-digit SOC codes associated with a higher odds of being an ALS participant were then subdivided into job-years worked 10 years prior to symptom onset (ALS participants) or survey consent (controls), 10–20 years prior to symptom onset/survey consent, and more than 20 years prior to symptom onset/survey consent, and subsequently inputted into adjusted logistic regression models to potentially identify important windows of exposure. Models were adjusted for age (quartiles), sex, and military service. The selected two-digit SOC codes were subdivided into six-digit SOC codes to see if particular types of jobs within the two-digit SOC code were driving the association.

### Joint analysis with exposure scores and SOC codes

Aggregated six-digit SOC codes were clustered based on the average exposure for the nine occupational exposure scores using the Euclidean distance as the distance metric to identify patterns of exposure by more granular occupational categorizations. Descriptive statistics for selected occupational exposure scores were tabulated for each two-digit SOC code. Associations between years worked in each two-digit SOC code and the selected occupational exposure scores were estimated using linear regression models and additive models, adjusted for age (quartiles), sex, and military service. The two-digit SOC codes associated with ALS/control status were subdivided into six-digit aggregated SOC codes, and then fit using linear regression models associating job-years worked within the six-digit SOC code and selected occupational exposure scores adjusted for age (quartiles), sex, and military service.

Analyses were performed for both occupational exposure score and duration-adjusted occupational exposure scores. Exposure scores were calculated using Excel and subsequent statistical analyses were performed in R.

## Results

### Participants

Between June 30, 2010 and February 12, 2020, completed surveys were received by 653 individuals: 381 from ALS and 272 from control participants (Table 1). This represents a 55% survey participation rate for the ALS group. Participation numbers by age category are shown in Table S2. ALS participants were slightly older than controls, 63.0 versus 61.2 years (*p* = 0.025) and had a smaller percentage of females, 45.1% versus 52.9% (*p* = 0.059). Educational attainment was statistically different as well with ALS participants having a larger percentage of high school or less education, 27.8% versus 8.5% (*p* < 0.001). ALS participants reflected a typical population with 18.4% meeting a definite El Escorial Diagnosis, 28.9% with bulbar onset, and 1.04 years median time from symptom onset to diagnosis.

**Table 1:** Participant Demographics

Covariate	Overall (*N* = 653)	ALS (*N* = 381)	Controls (*N* = 272)	*P* value
Age at survey consent (years)	62.5 (55.1–69.2)	63.0 (55.5–70.0)	61.2 (54.5–68.3)	0.025
Sex				0.059
Female	316 (48.4)	172 (45.1)	144 (52.9)	
Male	337 (51.6)	209 (54.9)	128 (47.1)	
Military Service				0.066
Neither	556 (85.1)	320 (84.0)	236 (86.8)	
Enlisted	89 (13.6)	61 (16.0)	28 (10.3)	
Missing	8 (1.2)	0 (0.0)	8 (2.9)	
Education				< 0.001
High School or less	129 (19.8)	106 (27.8)	23 (8.5)	
Some Postsecondary	200 (30.6)	123 (32.3)	77 (28.3)	
Bachelor’s Degree	168 (25.7)	87 (22.8)	81 (29.8)	
Graduate Degree	149 (22.8)	61 (16.0)	88 (32.4)	
Missing	7 (1.1)	4 (1.1)	3 (1.1)	
El Escorial Criteria				
Suspected		12 (3.2)		
Possible		42 (11.0)		
Probable, Lab Supported		105 (27.6)		
Probable		127 (33.3)		
Definite		95 (24.9)		
Onset Segment				
Bulbar		110 (28.9)		
Cervical		130 (34.1)		
Lumbar		139 (36.5)		
General		2 (0.5)		
Family History of ALS				
No		333 (87.4)		
Yes		33 (8.7)		
Unknown		12 (3.1)		
Missing		3 (0.8)		
Time Between Symptom Onset and Diagnosis (years)*		1.04 (0.66–1.77)		

Table of descriptive statistics for the study population. For continuous variables, Median (25th–75th percentile), and for categorical variables, *N* (%). *P* values for continuous and categorical variables correspond to analysis of variance tests and chi-squared tests, respectively

*Median and Interquartile Range calculated for 380 ALS participants, with one ALS case having a missing diagnosis date

### Occupational exposure scores

Participants provided self-reported occupational exposure histories for up to 4 jobs, the most recent (but before symptom onset for ALS), the one before the most recent, and the other 2 longest held jobs; a total of 1,867 unique jobs were reported after excluding jobs occurring after symptom onset (or after consent for controls). For jobs meeting the above criteria, ALS cases provided an average of 2.63 jobs and controls had 3.18 jobs. ALS participants had an average work duration of 31.0 years and control participants 28.6 years.

**Fig. 1:**
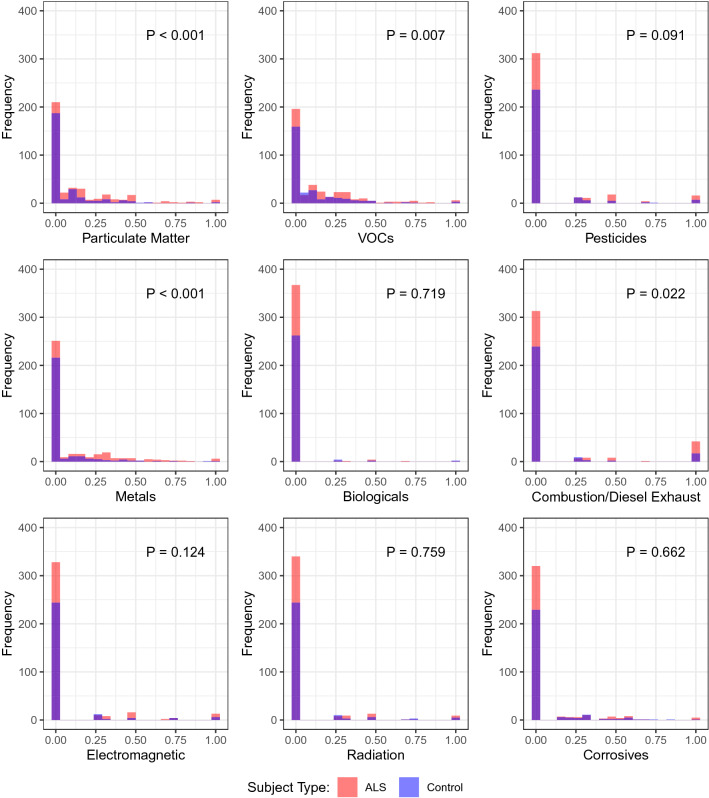
Occupational Exposure Score Histograms by ALS and Control. Overlapping histograms showing the distribution of occupational exposure scores for ALS (red) and control (blue) participants. Differences in occupational exposure scores between ALS and control participants were evaluated using permutation tests. *VOCs* volatile organic compounds; *P* p value

The median occupational exposure score and duration-adjusted occupational exposure score for all exposure types were 0, meaning that the questionnaire responses did not indicate PM, VOC or other exposures for most jobs and participants i.e., only a subset had occupational exposure based on survey responses (Tables 2, S3, Fig. 1). Tables S3 and 2 and Fig. 1 highlight the upper percentile of exposure scores between ALS and controls participants and shows significant differences in mean occupational exposure and duration-adjusted occupational exposure scores for ALS versus control participants for PM, VOCs, pesticides, metals, and combustion/diesel exhaust. For the occupational exposure scores and duration-adjusted occupational exposure scores, Spearman’s correlations were highest for PM and metals (*R* = 0.73), PM and combustion (*R* = 0.55), PM and VOCs (*R* = 0.53), and VOCs and metals (*R* = 0.60) (data not shown).

**Table 2:** Duration-adjusted occupational Exposure Scores

Exposure	Duration-adjusted occupational exposure score												*P*-value
	ALS (*N* = 381)						Control (*N* = 272)						
	*N*	Mean	SD	Q75	Q90	Q95	*N*	Mean	SD	Q75	Q90	Q95	
Particulate Matter (PM)	381	0.15	0.25	0.19	0.50	0.77	272	0.08	0.17	0.07	0.30	0.47	0.00
Volatile Organic Compounds (VOCs)	381	0.15	0.24	0.25	0.44	0.76	272	0.11	0.20	0.14	0.37	0.53	0.01
Pesticides	373	0.11	0.28	0.00	0.61	0.96	269	0.06	0.21	0.00	0.10	0.57	0.04
Metals	381	0.13	0.23	0.21	0.45	0.66	272	0.06	0.15	0.00	0.25	0.39	0.00
Biological Exposures	376	0.02	0.11	0.00	0.00	0.00	270	0.01	0.10	0.00	0.00	0.00	0.83
Combustion and Diesel Exhaust	379	0.14	0.33	0.00	1.00	1.00	270	0.08	0.26	0.00	0.11	1.00	0.02
Electromagnetic Exposure	381	0.10	0.27	0.00	0.48	0.93	272	0.07	0.22	0.00	0.00	0.80	0.12
Radiation	381	0.07	0.24	0.00	0.11	0.83	272	0.06	0.21	0.00	0.05	0.55	0.42
Corrosives	378	0.07	0.21	0.00	0.33	0.60	271	0.07	0.19	0.00	0.31	0.58	0.86

For all scores, minimum value is 0 and maximum is 1. Median for all scores is 0

*N* number, *SD* standard deviation, *Q* quartile

Differences in mean occupational exposure and duration-adjusted occupational exposure scores were seen when stratifying the population by sex (Tables S4 and S4a) and by education (Tables S5 and S5a). Overall, men and those with a high school or lower education are more likely to report occupational exposures.

### ALS status and occupational exposure

Differences in duration-adjusted exposure scores by ALS/control status were adjusted for age, sex, and military service using univariate logistic regression models to show how a one standard deviation increase in the occupational exposure score changes the odds of having ALS (Table 3). For the duration-adjusted occupational exposure scores, after adjusting for age, sex, and military service, PM (OR = 1.45, 95% CI 1.19–1.78, *p* < 0.001), VOCs (OR = 1.22, 1.02–1.45, *p* = 0.029), metals (OR = 1.48, 95% CI 1.21–1.82, *p* < 0.001), and combustion/diesel exhaust (OR = 1.20, 95% CI 1.01–1.43, *p* = 0.041) were all associated with increased ALS risk. In the multivariable logistic regression model, only occupational exposure to metals was significantly associated with ALS risk (OR = 1.56, 95% CI 1.11–2.20, *p* = 0.011), while unexpectedly, occupational exposure for corrosives was associated with a decreased risk (OR = 0.77, 95% CI 0.62–0.96, *p* = 0.021) (Table 3). Due to the correlated self-reported exposure scores, we utilized an adaptive elastic net model to account for mixtures, which selected PM (OR = 1.203), pesticides (OR = 1.015), metals (OR = 1.334), and corrosives (OR = 0.864). Overall, PM, VOCs, metals, and combustion/diesel exhaust were consistently identified as risk factors for ALS. As a sensitivity check, the model was rerun with age as a continuous variable and there were no significant differences compared to the model with age represented as quartiles. Similar models for the occupational exposure scores are presented in Table S6.

**Table 3:** ALS and control logistic regression models

Exposure score	Duration-adjusted occupational exposure scores						
	Univariate model			Multivariable model			AEN
	OR	95% CI	*P*-value	OR	95% CI	*P*-value	OR
Particulate matter (PM)	1.45	1.19–1.78	< 0.001	1.23	0.88–1.71	0.224	1.203
Volatile organic compounds (VOCs)	1.22	1.02–1.45	0.029	1.03	0.80–1.34	0.819	1.000
Pesticides	1.18	0.99–1.40	0.061	1.06	0.87–1.29	0.581	1.015
Metals	1.48	1.21–1.82	< 0.001	1.56	1.11–2.20	0.011	1.334
Biologicals	1.01	0.86–1.19	0.868	0.95	0.80–1.14	0.605	1.000
Combustion and diesel exhaust	1.20	1.01–1.43	0.041	1.02	0.82–1.27	0.851	1.000
Electromagnetic radiation	1.09	0.92–1.29	0.342	0.90	0.72–1.12	0.324	1.000
Radiation	1.07	0.91–1.26	0.391	0.97	0.81–1.17	0.757	1.000
Corrosives	1.01	0.86–1.19	0.910	0.77	0.62–0.96	0.021	0.864

Single exposure score logistic regression and multivariable logistic regression models where the outcome is ALS/control status, the variables of interest are the occupational duration-adjusted exposure scores, and the covariates are age, sex, and military service. The duration-adjusted occupational exposure scores are weighted by occupation duration

*AEN* adaptive elastic net, *OR* odds ratio, *CI* confidence interval

### Metals subcomponent scores

**Fig. 2:**
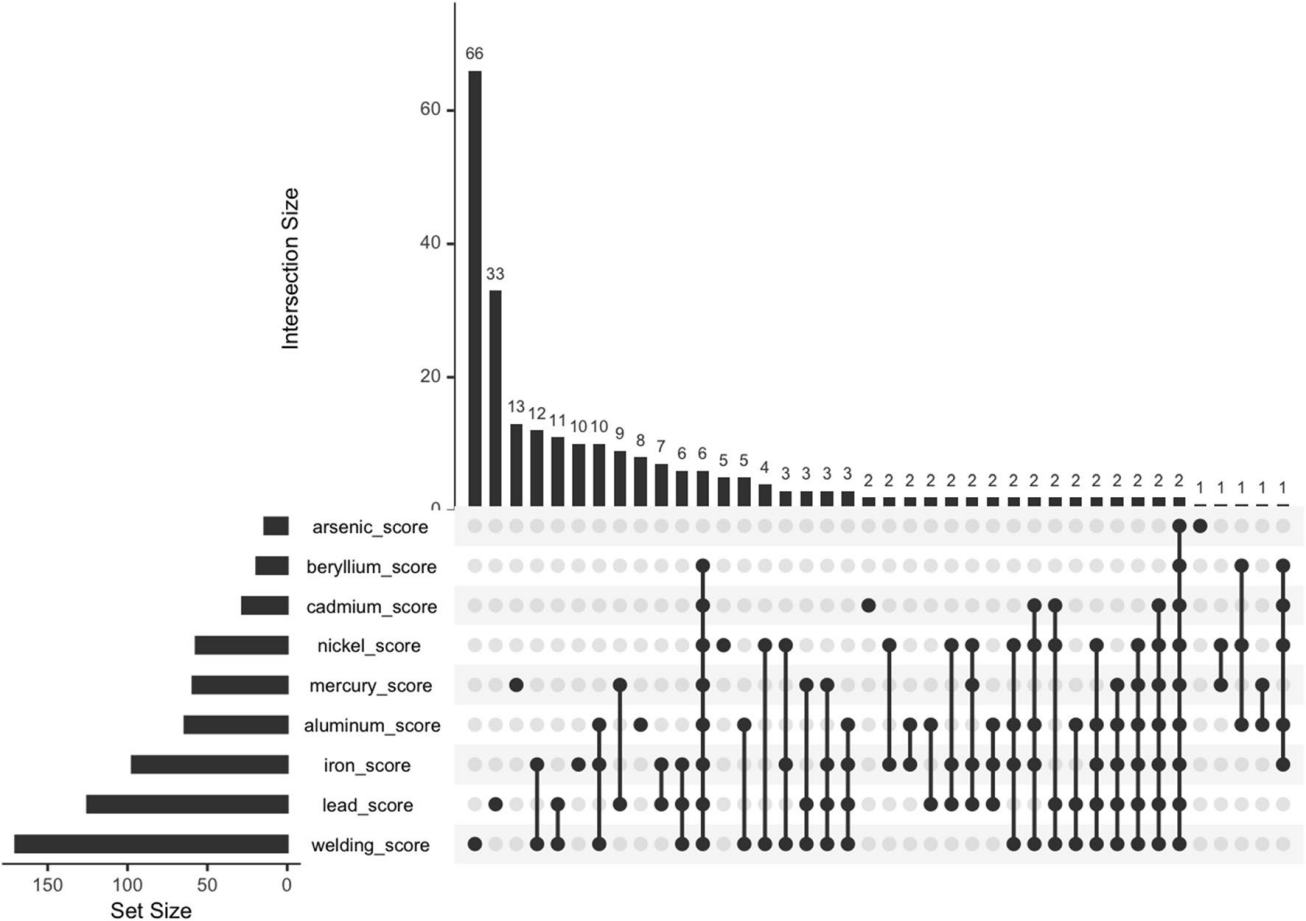
Self-reported occupational exposure responses to metal subcomponents. Upset plot showing the intersection of the subcomponent questions that comprise the metal score, for each job, for ALS and control participants combined

As metal exposures were the strongest risk factor across all models, we next examined the factors that determined these scores for both ALS and control participants. Figure 2 shows the overlap of individual metal subcomponent scores. The largest self-reported metal exposure, either alone or in a mixture, was to welding (*n* = 170), followed by lead (*n* = 125). Interestingly, 66 participants reported an isolated exposure to welding without other concurrent metals. We also evaluated the years worked for each job by each individual metal subcomponent score. For our cohort, this represented the period from 1950 to the present. Jobs with metal exposures spanned the full time period (1950–present), with the exception of arsenic where exposure was mostly reported from about 1970 to 2010 (Fig. S1). Lastly, we looked at each individual metal exposure on ALS risk and found that exposure to both iron (OR = 2.25, *p* = 0.006) and welding fumes (OR = 1.97, *p* = 0.003) were significant (Table S7).

### SOC codes

SOC codes are commonly used to assign individuals to occupations and exposure categories (Buckner-Petty et al. 2019). Of the universe of 465 SOC job codes, 374 were assigned to the 1867 occupations in our cohort; these were further reduced to 75 aggregated codes (73 codes were represented in ALS cases and 72 codes in controls (Table S8). These were further aggregated to the first 2 digits (representing industry) due to small counts in some codes. The average job-years reported for the four self-reported jobs meeting the inclusion criteria for ALS participants was 31.1 and for controls was 28.6; the difference in years is consistent with the slight difference in age in ALS versus control participants. The total number of ALS and controls and the respective job-years is shown in Table 4. Occupations with the highest percentage of job-years in the ALS group were “Production Occupations” (51-0000, 80.8%), “Installation, Maintenance, and Repair Occupations” (49-0000, 76.9%), and “Transportation and Material Moving Occupations” (53-0000, 75.9%). Compared to the study population (58% ALS and 42% control participants), there were significant differences in job-years worked between ALS and controls for four SOC codes (% job-year ALS, p value): 51-0000 “Production Occupations” (80.8%, *p* = 0.016); 11-0000 “Management Occupations” (49.6%, *p* = 0.002); 29-0000 “Healthcare Practitioners and Technical Occupations” (42.0%, *p* = 0.006); and 19-0000 “Life, Physical, and Social Science Occupations” (30.3%, *p* < 0.001).

**Table 4:** Job-Years and counts by ALS and control status

SOC Code	Occupational category	*N* (ALS)	Job-Years (ALS)	*N* (controls)	Job-Years (controls)	% Job-Years ALS	% ALS	*P*-value
51-0000	Production Occupations	78	1359.3	34	323.8	80.8	69.6	0.016
49-0000	Installation, Maintenance, and Repair Occupations	31	524.7	17	157.2	76.9	64.6	0.465
53-0000	Transportation and Material Moving Occupations	31	438.7	15	138.9	75.9	67.4	0.234
47-0000	Construction and Extraction Occupations	33	606.8	15	197.1	75.5	68.8	0.187
45-0000	Farming, Fishing, and Forestry Occupations	5	116.5	7	41.3	73.8	41.7	0.256
17-0000	Architecture and Engineering Occupations	40	709.4	24	339.0	67.7	62.5	0.529
41-0000	Sales and Related Occupations	64	849.9	55	458.2	65.0	53.8	0.353
35-0000	Food Preparation and Serving-Related Occupations	35	332.3	33	180.4	64.8	51.5	0.269
43-0000	Office and Administrative Support Occupations	108	1997.3	83	1088.6	64.7	56.5	0.608
37-0000	Building and Grounds Cleaning and Maintenance Occupations	27	294.4	20	172.0	63.1	57.4	0.884
25-0000	Education, Training, and Library Occupations	39	844.9	34	505.6	62.6	53.4	0.408
31-0000	Healthcare Support Occupations	18	232.6	15	144.5	61.7	54.5	0.725
39-0000	Personal Care and Service Occupations	16	252.1	14	174.1	59.1	53.3	0.584
21-0000	Community and Social Services Occupations	10	164.0	8	124.2	56.9	55.6	0.815
13-0000	Business and Financial Operations Occupations	32	535.6	32	439.7	54.9	50.0	0.205
23-0000	Legal Occupations	6	146.8	8	137.9	51.6	42.9	0.283
11-0000	Management Occupations	65	1158.0	78	1176.8	49.6	45.5	0.002
33-0000	Protective Service Occupations	8	120.0	11	151.5	44.2	42.1	0.167
27-0000	Arts, Design, Entertainment, Sports, and Media Occupations	15	231.8	21	297.7	43.8	41.7	0.061
29-0000	Healthcare Practitioners and Technical Occupations	27	566.7	39	781.2	42.0	40.9	0.006
55-0000	Military Occupations	8	30.1	8	55.6	35.1	50.0	0.614
15-0000	Computer and Mathematical Occupations	15	183.0	19	367.3	33.3	44.1	0.117
19-0000	Life, Physical, and Social Science Occupations	12	143.2	29	328.9	30.3	29.3	0.000

We next considered job-years worked in each two-digit SOC category on ALS risk (Table 5). In an unadjusted analysis, “Production Occupations” were associated with an increased ALS risk for every 5 years worked (OR = 1.25, 95% CI 1.09–1.42, *p* = 0.001), while three of the two-digit SOC categories were associated with a decreased ALS risk for every 5 years worked: “Computer and Mathematical Occupations” (OR = 0.81, 95% CI 0.67–0.98, *p* = 0.029), “Life, Physical, and Social Science Occupations” (OR = 0.72, 95% CI 0.56–0.92, *p* = 0.010), and “Healthcare Practitioners and Technical Occupations” (OR = 0.89, 0.80–0.99, *p* = 0.032). These effects remained statistically significant after adjusting for sex, age, and military service: ALS risk remained elevated for “Production Occupations” (OR = 1.22, 95% CI 1.07–1.40, *p* = 0.003), while risk was decreased for “Management Occupations” (OR = 0.90, 0.82–0.98, *p* = 0.015), “Computer and Mathematical Occupations” (OR = 0.78, 95% CI 0.65–0.94, *p* = 0.010), and “Life, Physical, and Social Science Occupations” (OR-0.73, 95% CI 0.57–0.94, *p* = 0.014). After correction for multiple comparisons, “Production Occupations” remained significant in unadjusted models (p = 0.024) and was marginally significant in adjusted models (*p* = 0.073). As a sensitivity check, the model was rerun with age as a continuous variable and there were no significant differences compared to the model with age represented as quartiles.

**Table 5:** Job-years worked with two-digit SOC codes associated with ALS Risk: single SOC code models

Two-Digit SOC Code	Description	Unadjusted				Adjusted			
		OR	95% CI	*P*-value	*P*-value (BH)	OR	95% CI	*P*-value	*P*-value (BH)
11-0000	Management Occupations	0.92	0.85–1.01	0.075	0.246	0.90	0.82–0.98	0.015	0.084
13-0000	Business and Financial Operations Occupations	0.97	0.86–1.10	0.664	0.770	0.95	0.83–1.08	0.408	0.587
15-0000	Computer and Mathematical Occupations	0.81	0.67–0.98	0.029	0.187	0.78	0.65–0.94	0.010	0.084
17-0000	Architecture and Engineering Occupations	1.09	0.95–1.25	0.209	0.474	1.07	0.93–1.23	0.373	0.572
19-0000	Life, Physical, and Social Science Occupations	0.72	0.56–0.92	0.010	0.111	0.73	0.57–0.94	0.014	0.084
21-0000	Community and Social Services Occupations	0.99	0.77–1.26	0.915	0.945	0.99	0.77–1.27	0.947	0.947
23-0000	Legal Occupations	0.95	0.77–1.18	0.670	0.770	0.94	0.76–1.17	0.590	0.646
25-0000	Education, Training, and Library Occupations	1.03	0.93–1.15	0.552	0.746	1.04	0.93–1.16	0.478	0.611
27-0000	Arts, Design, Entertainment, Sports, and Media Occupations	0.91	0.77–1.07	0.248	0.474	0.92	0.79–1.09	0.341	0.561
29-0000	Healthcare Practitioners and Technical Occupations	0.89	0.80–0.99	0.032	0.187	0.91	0.82–1.01	0.090	0.319
31-0000	Healthcare Support Occupations	1.04	0.81–1.33	0.758	0.830	1.11	0.86–1.42	0.434	0.587
33-0000	Protective Service Occupations	0.88	0.68–1.14	0.331	0.544	0.86	0.67–1.12	0.260	0.543
35-0000	Food Preparation and Serving-Related Occupations	1.10	0.86–1.41	0.437	0.670	1.14	0.88–1.47	0.318	0.561
37-0000	Building and Grounds Cleaning and Maintenance Occupations	1.06	0.85–1.32	0.623	0.770	1.07	0.85–1.34	0.552	0.635
39-0000	Personal Care and Service Occupations	1.01	0.83–1.23	0.945	0.945	1.03	0.85–1.26	0.747	0.781
41-0000	Sales and Related Occupations	1.08	0.94–1.23	0.267	0.474	1.07	0.94–1.23	0.296	0.561
43-0000	Office and Administrative Support Occupations	1.07	0.98–1.15	0.124	0.316	1.08	0.99–1.17	0.085	0.319
45-0000	Farming, Fishing, and Forestry Occupations	1.14	0.79–1.64	0.478	0.687	1.13	0.78–1.63	0.531	0.635
47-0000	Construction and Extraction Occupations	1.16	0.99–1.36	0.072	0.246	1.14	0.96–1.34	0.128	0.319
49-0000	Installation, Maintenance, and Repair Occupations	1.21	0.99–1.47	0.059	0.246	1.16	0.95–1.41	0.139	0.319
51-0000	Production Occupations	1.25	1.09–1.42	0.001	0.024	1.22	1.07–1.40	0.003	0.073
53-0000	Transportation and Material Moving Occupations	1.19	0.97–1.46	0.096	0.276	1.18	0.96–1.44	0.120	0.319
55-0000	Military Occupations	0.61	0.26–1.46	0.268	0.474	0.42	0.13–1.29	0.129	0.319

Single exposure score logistic regression models where the outcome is ALS/control status, the variables of interest are the number of job-years worked within two-digit SOC codes, and the covariates are age, sex, and military service. Interpretation of odds ratios (OR) correspond to 5 additional years worked within the respective SOC code

*CI* confidence interval, *BH* Bejamini–Hochberg

### Exposure scores and SOC codes

**Fig. 3:**
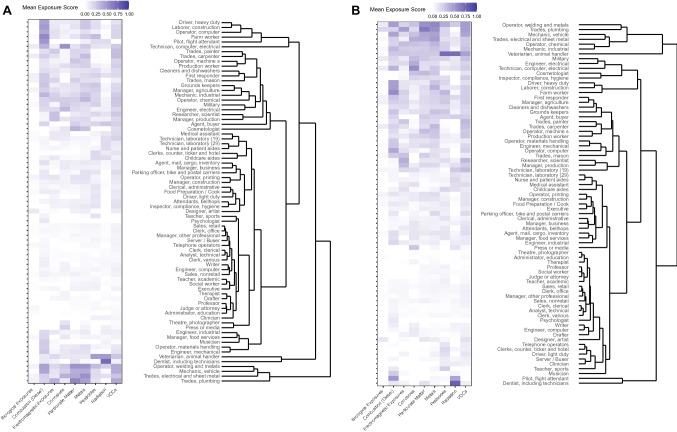
Occupational exposure scores by aggregated SOC clusters. Dendrograms of the standard occupational classification (SOC) codes by **A** occupational exposure score and **B** duration-adjusted occupational exposure scores

We explored the relationships of the occupational exposure scores to SOC codes via a dendrogram using the occupational exposure score and duration-adjusted occupational exposure scores (Fig. 3A, B). The occupational exposure score dendrogram showed a small cluster (bottom of plot) including “Trades, plumbing,” “Trades, electrical and sheet metal,” “Mechanic, vehicle,” and “Operator, welding and metals.” The duration-adjusted occupational exposure score dendrogram showed a small cluster (top of plot) containing “Veterinarian, animal handler,” “Mechanic, industrial,” “Operator, chemical,” “Trades, electrical and sheet metal,” “Mechanic, vehicle,” “Trades, plumbing,” and “Operator, welding and metals.” These clusters were overall characterized by higher exposure scores. The lack of other clear smaller clusters highlights that SOC codes alone do not capture the full range of exposures that occur in the occupational setting, and that self-reported details (e.g., in the questionnaire) can be highly informative.

Because occupational exposure to metals was significantly associated with ALS risk in all models (Table 6), we next compared the metals occupational exposure scores by SOC code. The five occupations with the highest mean metals occupational exposure scores were: 49-0000 “Installation, Maintenance, and Repair Occupations;” 51-0000 “Production Occupations;” 55-0000 “Military Occupations;” 47-0000 “Construction and Extraction Occupations;” and 17-0000 “Architecture and Engineering Occupations.” Several occupations with overall lower scores had a large range of exposure scores, e.g., 11-0000 “Management Occupations” had scores ranging from 0 to 1.00. This reflects the large range of tasks and activities that can fall into the two-digit SOC classifications, e.g., some managers work exclusively in offices with no exposure to metals, while managers in services, trades or production settings may experience relatively high exposure.

**Table 6:** Metals occupational exposure scores associated with SOC codes

Two-Digit SOC	Description	*N*	Mean	SD	Min	Q25	Q50	Q75	Max
49-0000	Installation, Maintenance, and Repair Occupations	55	0.31	0.36	0	0	0.33	0.48	1.00
51-0000	Production Occupations	148	0.25	0.32	0	0	0.00	0.46	1.00
55-0000	Military Occupations	17	0.25	0.33	0	0	0.00	0.33	1.00
47-0000	Construction and Extraction Occupations	63	0.19	0.30	0	0	0.00	0.33	1.00
17-0000	Architecture and Engineering Occupations	100	0.15	0.28	0	0	0.00	0.18	1.00
33-0000	Protective Service Occupations	23	0.15	0.18	0	0	0.00	0.28	0.61
45-0000	Farming, Fishing, and Forestry Occupations	12	0.12	0.20	0	0	0.00	0.13	0.61
19-0000	Life, Physical, and Social Science Occupations	52	0.08	0.19	0	0	0.00	0.00	0.67
11-0000	Management Occupations	191	0.08	0.22	0	0	0.00	0.00	1.00
37-0000	Building and Grounds Cleaning and Maintenance Occupations	50	0.08	0.23	0	0	0.00	0.00	1.00
53-0000	Transportation and Material Moving Occupations	54	0.06	0.16	0	0	0.00	0.00	0.72
25-0000	Education, Training, and Library Occupations	107	0.05	0.17	0	0	0.00	0.00	0.78
31-0000	Healthcare Support Occupations	46	0.05	0.15	0	0	0.00	0.00	0.56
13-0000	Business and Financial Operations Occupations	86	0.04	0.15	0	0	0.00	0.00	1.00
21-0000	Community and Social Services Occupations	33	0.04	0.10	0	0	0.00	0.00	0.33
27-0000	Arts, Design, Entertainment, Sports, and Media Occupations	47	0.03	0.11	0	0	0.00	0.00	0.44
43-0000	Office and Administrative Support Occupations	303	0.02	0.11	0	0	0.00	0.00	1.00
29-0000	Healthcare Practitioners and Technical Occupations	116	0.02	0.09	0	0	0.00	0.00	0.61
35-0000	Food Preparation and Serving-Related Occupations	85	0.02	0.13	0	0	0.00	0.00	0.89
41-0000	Sales and Related Occupations	153	0.02	0.08	0	0	0.00	0.00	0.61
15-0000	Computer and Mathematical Occupations	50	0.01	0.06	0	0	0.00	0.00	0.33
23-0000	Legal Occupations	20	0.00	0.00	0	0	0.00	0.00	0.00
39-0000	Personal Care and Service Occupations	29	0.00	0.00	0	0	0.00	0.00	0.00

*N* number, *SD* standard deviation, *Min* minimum, *Q* quartile, *Max* maximum

Linear models adjusted for age, sex, and military service were next developed to understand the association between duration of each SOC code and metals duration-adjusted occupational exposure. For every 5 years of work, the standard deviation changes in metals duration-adjusted occupational exposure scores were as follows: 13-0000 “Business and Financial Operations Occupations” (beta =  − 0.08, 95%CI − 0.14- − 0.02 *p* = 0.006); 15-0000 “Computer and Mathematical Occupations” (*β* = − 0.08, − 0.16 to − 0.01, *p* = 0.037); 37-0000 “Building and Grounds Cleaning and Maintenance Occupations” (*β* = 0.12, 95% CI 0.02–0.22, *p* = 0.020); 41-0000 “Sales and Related Occupations” (*β* =  − 0.09, 95%CI − 0.15- − 0.04, *p* = 0.002); 47-0000 “Construction and Extraction Occupations” (*β* = 0.14, 95% CI 0.08–0.20, *p* < 0.001); 49-0000 “Installation, Maintenance, and Repair Occupations” (*β* = 0.15, 0.07–0.22, *p* < 0.001); and 51-0000 “Production Occupations” (*β* = 0.18, 0.14–0.23, *p* < 0.001) (Table 7). A similar analysis for the metal occupational exposure score is presented in Table S9. Unsurprisingly, these data indicate that occupational exposure to metals is more likely to be reported in “Construction and Extraction,” “Installation, Maintenance, and Repair,” and “Production Occupations.”

**Table 7:** Metal duration-adjusted occupational exposure score association with SOC codes

Two-Digit SOC Code	Description	Metal duration-adjusted occupational exposure score			
		*Β*	95% LCL	95% UCL	*P*-value
11-0000	Management Occupations	– 0.03	– 0.07	0.01	0.173
13-0000	Business and Financial Operations Occupations	– 0.08	– 0.14	– 0.02	0.006
15-0000	Computer and Mathematical Occupations	– 0.08	– 0.16	– 0.01	0.036
17-0000	Architecture and Engineering Occupations	0.01	– 0.05	0.07	0.687
19-0000	Life, Physical, and Social Science Occupations	0.01	– 0.08	0.11	0.780
21-0000	Community and Social Services Occupations	– 0.04	– 0.16	0.07	0.456
23-0000	Legal Occupations	– 0.07	– 0.17	0.04	0.207
25-0000	Education, Training, and Library Occupations	– 0.01	– 0.06	0.04	0.758
27-0000	Arts, Design, Entertainment, Sports, and Media Occupations	– 0.02	– 0.09	0.06	0.652
29-0000	Healthcare Practitioners and Technical Occupations	– 0.01	– 0.05	0.04	0.832
31-0000	Healthcare Support Occupations	0.02	– 0.10	0.13	0.761
33-0000	Protective Service Occupations	0.08	– 0.04	0.20	0.173
35-0000	Food Preparation and Serving-Related Occupations	– 0.04	– 0.15	0.07	0.443
37-0000	Building and Grounds Cleaning and Maintenance Occupations	0.12	0.02	0.22	0.020
39-0000	Personal Care and Service Occupations	– 0.04	– 0.13	0.05	0.391
41-0000	Sales and Related Occupations	– 0.09	– 0.15	– 0.04	0.002
43-0000	Office and Administrative Support Occupations	– 0.03	– 0.07	0.01	0.135
45-0000	Farming, Fishing, and Forestry Occupations	0.01	– 0.13	0.14	0.937
47-0000	Construction and Extraction Occupations	0.14	0.08	0.20	0.000
49-0000	Installation, Maintenance, and Repair Occupations	0.15	0.07	0.22	0.000
51-0000	Production Occupations	0.18	0.14	0.23	0.000
53-0000	Transportation and Material Moving Occupations	– 0.05	– 0.13	0.03	0.213
55-0000	Military Occupations	– 0.02	– 0.32	0.28	0.893

Single two-digit SOC logistic regression models and generalized additive models where the outcome is the metal duration-adjusted occupational exposure score, the variables of interest are the number of job-years worked within each two-digit SOC code, and the covariates are age, sex, and military service. Interpretation of coefficient is in in terms of 5 year increments corresponding to standard deviation changes in occupational metal score

*LCL* lower confidence limit, *UCL* upper confidence limit

## Discussion

Understanding non-genetic ALS risk factors is critically important to identify factors that increase disease risk, the underlying mechanisms, and potential preventative strategies. Our analysis of occupational exposures, based on a comprehensive survey and job classification coding, found that self-reported exposure to metals and a history of working in “Production Occupations” (SOC 51-0000)—which includes production workers, welders and metal, machine, printing, and chemical operators—increased ALS risk. ALS risk also increased with self-reported occupational exposures to particulate matter, volatile organic compounds, pesticides, metals, and combustion/diesel exhaust in univariate and adaptive elastic net models.

Our findings are consistent with other published reports, which demonstrate that survey-based tools (Morahan and Pamphlett 2006; Bonvicini et al. 2010) and structured interviews (Malek et al. 2014; McGuire et al. 1997) are informative for examining ALS environmental risk factors. Studies on occupational risk factors differ in terms of design, job and exposure ascertainment, and other factors, and results are not always consistent. Table 8 presents the key findings from several occupational exposure studies and their alignment with the current report. A large population-based study in the Netherlands, Ireland, Apulia, Lombardy, and Piedmont and Valle d’Aosta in Italy including 1157 ALS participants showed that occupational exposures to silica, asbestos, organic dust, contact with animals or fresh animal products, endotoxins, polycyclic aromatic hydrocarbons and diesel motor exhaust were all associated with an increased ALS risk (Visser et al. 2019). Our univariate analyses provided consistent findings for exposure to particulate matter (which includes exposures to silica) as well as combustion products and diesel exhaust. A study in Pennsylvania, United States showed that occupational exposures to metals and pesticides increased ALS risk (Malek et al. 2014). A study in Australia showed that men who worked with metals, chemicals/solvents, and herbicides/pesticides and women who reported a higher exposure to chemicals/solvents had a higher risk of ALS (Pamphlett 2012). Another Australian study showed that male technicians and trade workers, machinery operators and drivers, and laborers had an increased risk of ALS and that truck driving as an occupation was associated with a higher ALS risk (Pamphlett and Rikard-Bell 2013).

**Table 8:**
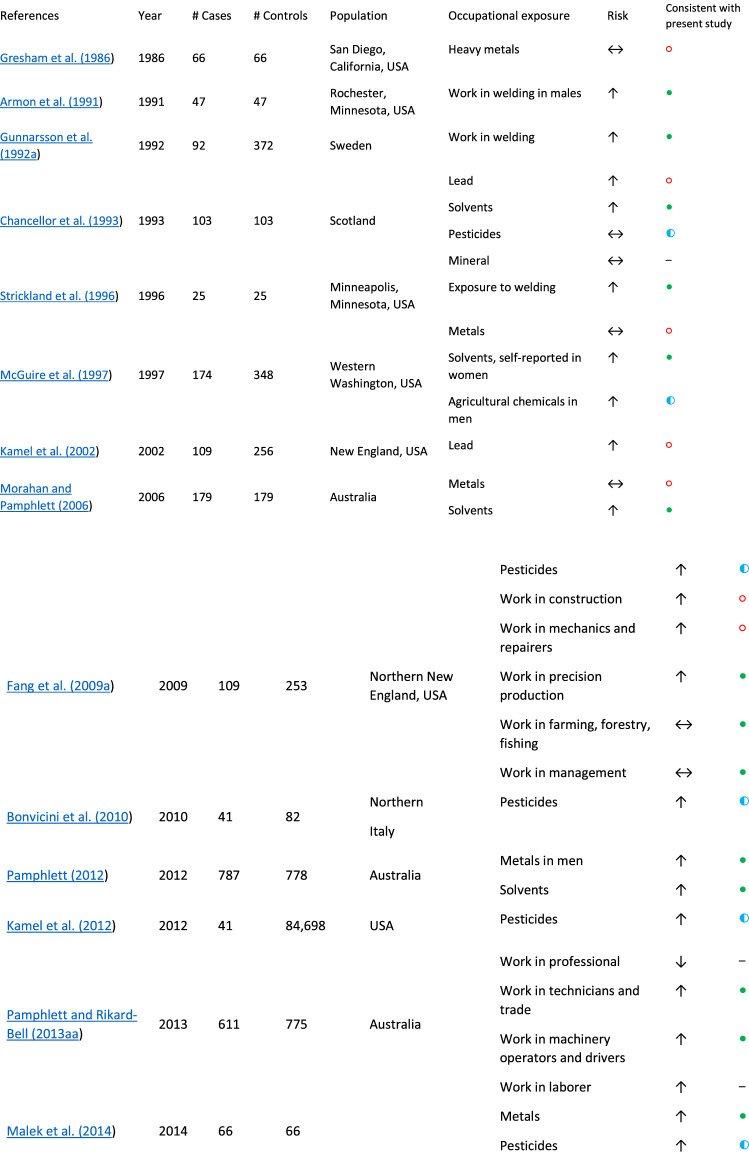

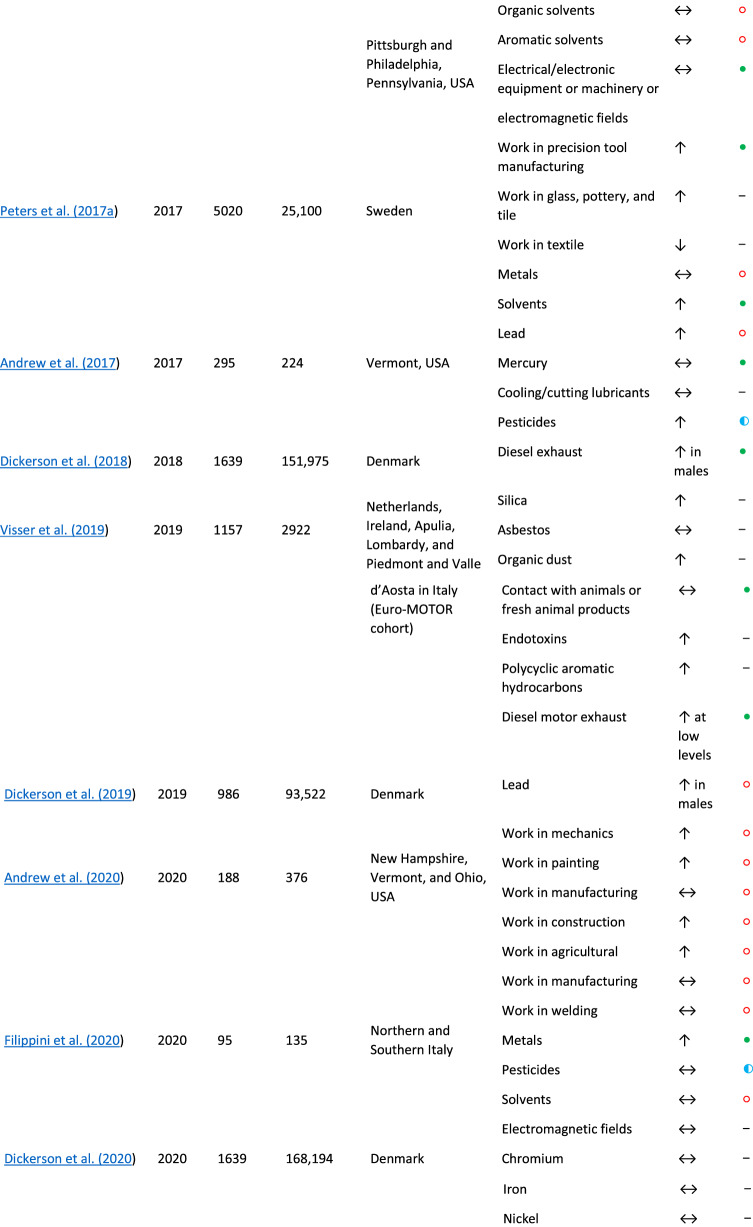
Summary of ALS occupational case/control studies (by publication year)

↑ increased risk, ↓ decreased risk, ↔  no difference in risk, *NE* not evaluated,  risk consistent with current study,  risk partially consistent with current study,  risk not consistent with current study, – not evaluated

^a^Due to the number of exposures analyzed in the referenced study, only outcomes highlighted in the present study are listed

The multivariable models showed that self-reported occupational metals exposure was most strongly linked to ALS risk. This could be related to the type of correlated exposures experienced in certain occupations where participants with higher metals occupational exposure scores also reported higher exposures to particulate matter, volatile organic compounds, and corrosives. This is unsurprising as workers are often exposed to mixtures, particularly in certain trades, production/manufacturing, and service industries (Mixed Exposures Research Agenda 2004). In some respects, this is similar to our findings for mixtures of persistent organic pollutants in our cohort (Goutman et al. 2019)—participants are exposed to polychlorinated biphenyls, brominated flame retardants, and organochlorine pesticides, for example, yet pesticides alone carry the highest risk. In this present study, metals may be the most critical component of a larger occupational exposure mixture. This should lead us to focus on the types of mixtures and resulting injuries to the central nervous system in future research focused on understanding ALS pathogenesis. This mixture effect led to the adaptive elastic net analysis that showed overall consistency with the univariate models, again with occupational metals exposure carrying the strongest association.

Among the individual metals/tasks, iron and welding fume exposure were the most significant; these exposures were also among the most common. Welding has been linked to ALS in prior studies (Gunnarsson et al. 1992; Strickland et al. 1996; Armon et al. 1991). Dickerson et al. did not find iron to be a risk factor in Denmark (Dickerson et al. 2020). Although we did not find a significant association between occupational lead exposure and ALS, this has been shown in other survey-based studies, e.g., Dickerson et al. used a job-exposure matrix in a Danish population and found that occupational exposure led to increased ALS risk (Dickerson et al. 2019), and both Kamel et al. in a New England population (Kamel et al. 2002) and Chancellor et al. in a Scottish population (Chancellor et al. 1993) found that self-reported occupational exposure to lead increased ALS risk. Of note, other studies, like ours, report no association with occupational lead exposure (Gunnarsson et al. 1992; Gresham et al. 1986).

The questionnaire data included 66 jobs for welding where no other metal exposure was reported (Fig. 2). Since welding requires the use of metals (OSHA 2021), this may mean that participants were not aware of their exposure. Plotting metal exposure by years the on the job, we saw that essentially all metals (except for arsenic) continue to be reported to the present, suggesting that metal exposure continues to be an actionable risk factor. Our finding that welding and iron exposure are ALS risk factors may indicate that workers are exposed to other metals without their knowledge, that welding and iron exposure represent surrogates for some other exposure, or that mixtures of metals play a greater role on ALS risk compared to an individual metal, similar to what we have seen in our analysis of teeth (Figueroa-Romero et al. 2020).

Among the job codes, “Production Occupations” was most strongly associated with ALS risk. This is a diverse category that includes production workers, welders, and metals, machine, printing, and chemical operators. ALS risk is highly variable in this group, potentially reflecting the variability of occupational exposures to metals in this code. For example, in this job code, most participants had a zero metal exposure score, while a small group of participants accounted for the bulk of exposure. High metals occupational exposure scores also occurred for workers in other job codes, e.g., “Building and Grounds Cleaning and Maintenance Occupations,” “Construction and Extraction Occupations,” and “Installation, Maintenance, and Repair Occupations,” demonstrating the value of complementing the job codes with personal-level exposure data. Our findings are consistent with other studies. Andrew et al. found that working in mechanics, painting, or construction increased ALS risk (Andrew et al. 2020), and, in a separate study, that working in construction, manufacturing, mechanical, military, or painting occupations increased ALS risk (Andrew et al. 2017). In parallel, Fang et al. also found that construction and precision metal workers were at an increased ALS risk (Fang et al. 2009).

We examined the job titles and tasks linked to the metals occupational exposure scores, and graded each exposure as probable, possible or unlikely based on this information. For example, lead exposure was reported by individuals who had worked in construction (e.g., builders, painters, pipefitters, electricians, plumbers, remodelers, handymen), boat restorers, maintenance workers, some automotive shop and factory workers, some workers in steel and metal industries (metallurgist, welders), and X-ray technicians. In these industries, lead exposure can occur from lead in paint, plumbing, solder, and other materials. However, self-reported lead exposure for a subset (17%) of workers did not appear concordant with reported job titles and tasks (e.g., some truckers and property managers), while a smaller subset (9%) did not report lead exposure although it may have occurred (some skilled trades). Workers reporting mercury exposure included dentists and dental staff, and some production, waste and engineering workers; this is reasonable given mercury in dental amalgam and some (older) electrical switches and other equipment. Again, a subset (20%) of self-reported mercury exposures seemed unlikely, for example, education and most metal workers. For cadmium exposure, a wide range of workers reported exposure, e.g., researchers, engineers, automotive workers, painters, and some production and metal workers. While cadmium has been used in metal plating, paints and coatings, the likelihood of this exposure is difficult to assess based on the survey data. For arsenic, self-reported exposure was reported by only six workers. Again, exposure is difficult to confirm; arsenic is present in some agricultural chemicals and pressure treated wood, but no farmers, construction, building or grounds workers reported this exposure. While any survey will have issues of accuracy, omissions, recall bias, etc., our results suggest a reasonable degree of consistency for the more common and recognized metal exposures (e.g., lead), but also the challenge for other metals. Arsenic exposure, for example, might be better handled by questions to construction and agricultural workers such as “did you “handle treated wood?,” although this would increase the complexity and length of the survey.

Our findings add to a growing literature of potential occupational ALS risk factors. Importantly, it should be noted that all studies are not uniform. For example, a nested case–control study in Sweden did not show that occupational exposure to metal was an ALS risk factor (Peters et al. 2017). Further, a study of 1 million participants from a cancer prevention cohort study did not show an increased ALS risk among farmers, electricians, and welders (Weisskopf et al. 2005). Like the large ALS occupational risk assessment in the prospective Netherlands Cohort Study and the Western Washington study, we find that ALS participants have a lower educational attainment compared to controls (McGuire et al. 1997; Koeman et al. 2017). However, while we find occupational exposures to metals increase ALS risk, the Netherlands study did not. An important difference is that we used individual reporting as opposed to only assigning risk based on a job-exposure matrix. This is a strength for our study as reported exposures are not uniform across each job code.

Also of note, independent of case status, men and those with high school or lower secondary education report higher occupational exposures. These groups may require public health attention to lessen exposure risks. McGuire et al. (McGuire et al. 1997) found in their ALS case and control cohort that men had higher exposures to agricultural chemicals. Our results were partially consistent with pesticide exposure showing a small risk in the adaptive elastic net model, although those working in farming, fishing, and forestry did not have a significant association, which could be due to incomplete case capture, or changes in in occupational exposures since the publication of that study in 1997. These findings should be interpreted cautiously as we have previously shown higher levels of organochlorine pesticides in ALS participants. We also did not find that occupational exposure to electromagnetic radiation increases ALS risk, which could be due to the low number of individuals reporting this exposure in our cohort, most of whom were health care workers. A recent meta-analysis suggests that electromagnetic radiation exposure slightly increases ALS risk (Jalilian et al. 2021), although reports included in the meta-analysis were mixed, indicating that the risk is not uniform across all studies.

In prior studies, including our own, pesticide exposure was identified as an ALS risk factor (Al-Chalabi and Hardiman 2013; Su et al. 2016; Kamel et al. 2012). Specifically, an exposure history to agricultural chemicals increased ALS risk for individuals in western Washington State (McGuire et al. 1997). Bonvicini and colleagues used a questionnaire to show that pesticide exposure increased ALS risk in a northern Italian population (Bonvicini et al. 2010). Morahan and colleagues used a questionnaire to show that ALS risk in Australia was associated with solvent/chemical exposure, herbicide/pesticide exposure, and industrial herbicide/pesticide exposure (Morahan and Pamphlett 2006). Although pesticide exposure is a small risk factor in the adaptive elastic net logistic regression model using the duration-adjusted occupational exposure scores, this finding is not present in other models. This is possibly because this exposure may largely be occurring outside the workplace, and because we captured relatively few participants with occupational pesticide exposure, such as individuals in the farming industry. Thus, further work exploring exposure across multiple settings (occupational and residential) is needed. Additionally, prospective cohorts of individuals that have higher exposures to pesticides would be beneficial.

We used a combined approach of self-reported occupational exposures and occupational histories, augmented by expert assessment, to identify ALS occupational risk factors. Occupational histories, self-reported exposure assessments, and expert assessment are the main strategies used in retrospective case–control studies examining occupational exposures (Ge et al. 2018; Teschke et al. 2002). All techniques are especially challenging when retrospectively identifying disease risk factors with a long latency period (Ge et al. 2018). Occupational histories—a listing of job titles and responsibilities—have several limitations impacting reliability, especially when the job title does not reflect the work performed (Teschke et al. 2002). Nonetheless, occupational histories can help identify certain at-risk occupations, which in turn can highlight mixtures of chemicals typically used in that occupation without zeroing in on a specific chemical or exposure (Teschke et al. 2002). Generic job-exposure matrices (JEMs) share limitations of occupational histories by not capturing a full range of exposures or homing in on a specific risk (Teschke et al. 2002). Self-reported exposures, subject to recall bias, can outperform JEMs as they provide individualized data on job activities. With self-reported exposures there is no gold standard for comparison, e.g., participants may not know the names of chemicals to which they were exposed (Teschke et al. 2002). The performance of these techniques can improve by both focusing on a specific set of exposures and complementing expert assessment with self-reported exposures, the study design with the highest accuracy (Ge et al. 2018; Teschke et al. 2002). Thus, this was our approach.

Outside of ALS, this study has other important findings. Automated systems that assign SOC codes to occupations are a useful tool, especially when a large number of occupations require classification. However, despite using two separate systems from NIH and CDC, additional input from exposure scientists was needed, consistent with other findings related to these auto-coding systems (Buckner-Petty et al. 2019). We also found that the SOC coding is insufficient to account for exposures, especially in certain occupations where the same job code can encompass very diverse occupational settings.

This study has several strengths. First, we captured a large number of participants in Michigan, a diverse state with an historical agricultural and industrial legacy. Second, the questionnaire obtained detailed self-reported information on exposures. We identified at-risk occupations via SOC coding and showed that complementing SOC codes with self-reported exposures is meaningful, thus addressing the variability of exposures across a job code. Hand curation by exposure scientists provided further refinement of the automated SOC coding. Overall, our approach combining expert assessment with self-reported exposures and targeted automated SOC codes was consistent with best practices identified in the literature (Ge et al. 2018).

This study also has limitations. Selection bias is possible as not all persons with ALS seen in our clinic enrolled in this study. The participation rate of 55% is consistent with other large ALS cohorts, including the National ALS Registry (completion rate of 43.6–49.2%) (Bryan et al. 2016). Further, the control population was based on altruism. While our control population was more highly educated compared to controls, this could represent a true difference, especially as polygenic factors associated with higher educational attainment are associated with a lower ALS risk (Bandres-Ciga et al. 2019). There is no gold standard for self-reported exposure assessment, and recall bias may influence results. As military service is a recognized ALS risk factor (Al-Chalabi and Hardiman 2013), we elected to adjust models for military service history, but did not include self-reported military exposures in the exposure scores. Also, while only a subset of individuals experienced work-related exposures to particulate matter (PM), volatile organic compounds (VOCs), metals and other contaminants, this is expected and reflects the contemporary distribution of job types. These are still important findings as the identification of environmental ALS risk factors, even in small groups, helps identify modifiable factors that could be used to better understand how to prevent disease in certain population groups. We do not differentiate between full- and part-time employment and only considered job-years in the analysis. This should not result in exposure misclassification as exposure scores were based on participants’ responses to questions relevant to exposures, e.g., do they work with specific chemicals? First, most of these questions were “yes/no”, and quantification of exposure (like a dose or concentration) from such survey questions is not possible. Second, the survey questions were repeated for up to four different jobs, from which we calculated an overall (duration-adjusted) occupational exposure score. The survey responses suggest that most participants had had several jobs that tended to be similar, e.g., staying in the service or educational sector, thus likely diminishing the potential effect of a part-time versus full-time position. Third, in most cases, the job title, descriptors, and survey questions suggested that most individuals described full-time jobs, although we do not have direct evidence. Finally, we believe that differences in exposure contrast across jobs in the different sectors (e.g., as a mechanic, food preparation, or office worker) likely exceed the difference that might result due to whether an individual works 20, 30 or 40 h. It is important to note too that analyses that did not consider duration of each occupation showed similar results. Finally, we focused on occupational exposures, whereas non-occupational exposures do occur and may also contribute to ALS risk.

## Conclusion

Self-reported occupational exposures to particulate matter, volatile organic compounds, metals, and combustion and diesel exhaust are identified as ALS risk factors. The greatest risks were self-reported occupational metals exposure among exposure types, and production occupations among job codes. Overall, these data provide important insights into the occupational exposures and settings that increase ALS risk. Further investigations are encouraged to understand the mechanisms that lead to this increase in risk. Additionally, these data may be informative for ALS prevention strategies designed to limit exposures, especially for people most at risk of developing ALS.

## Supplementary Information

Below is the link to the electronic supplementary material.

Supplementary file1 (DOCX 959 KB)

## Data Availability

Sharing of non-identifiable data will be considered at the reasonable request of a qualified investigator.

## References

[CR1] Al-Chalabi A, Hardiman O (2013). The epidemiology of ALS: a conspiracy of genes, environment and time. Nat Rev Neurol.

[CR2] Al-Chalabi A, Calvo A, Chio A (2014). Analysis of amyotrophic lateral sclerosis as a multistep process: a population-based modelling study. Lancet Neurol.

[CR3] Andrew AS, Caller TA, Tandan R (2017). Environmental and occupational exposures and amyotrophic lateral sclerosis in New England. Neurodegener Dis.

[CR4] Andrew AS, Bradley WG, Peipert D (2020). Risk factors for amyotrophic lateral sclerosis: a regional United States case-control study. Muscle Nerve.

[CR5] Armon C, Kurland LT, Daube JR, O'Brien PC (1991). Epidemiologic correlates of sporadic amyotrophic lateral sclerosis. Neurology.

[CR6] ATSDR (2000) Taking an exposure history. In: Case studies in environmental medicine

[CR7] Bandres-Ciga S, Noyce AJ, Hemani G (2019). Shared polygenic risk and causal inferences in amyotrophic lateral sclerosis. Ann Neurol.

[CR8] Bonvicini F, Marcello N, Mandrioli J, Pietrini V, Vinceti M (2010). Exposure to pesticides and risk of amyotrophic lateral sclerosis: a population-based case-control study. Ann Ist Super Sanita.

[CR9] Bryan L, Kaye W, Antao V, Mehta P, Muravov O, Horton DK (2016). Preliminary results of National Amyotrophic Lateral Sclerosis (ALS) registry risk factor survey data. PLoS ONE.

[CR10] Buckner-Petty S, Dale AM, Evanoff BA (2019). Efficiency of autocoding programs for converting job descriptors into standard occupational classification (SOC) codes. Am J Ind Med.

[CR11] Chancellor AM, Slattery JM, Fraser H, Warlow CP (1993). Risk factors for motor neuron disease: a case-control study based on patients from the Scottish Motor Neuron Disease Register. J Neurol Neurosurg Psychiatry.

[CR12] Dickerson AS, Hansen J, Gredal O, Weisskopf MG (2018). Amyotrophic lateral sclerosis and exposure to diesel exhaust in a Danish cohort. Am J Epidemiol.

[CR13] Dickerson AS, Hansen J, Specht AJ, Gredal O, Weisskopf MG (2019). Population-based study of amyotrophic lateral sclerosis and occupational lead exposure in Denmark. Occup Environ Med.

[CR14] Dickerson AS, Hansen J, Gredal O, Weisskopf MG (2020). Study of occupational chromium, iron, and nickel exposure and amyotrophic lateral sclerosis in Denmark. Int J Environ Res Public Health.

[CR15] Fang F, Quinlan P, Ye W (2009). Workplace exposures and the risk of amyotrophic lateral sclerosis. Environ Health Perspect.

[CR16] Figueroa-Romero C, Mikhail KA, Gennings C (2020). Early life metal dysregulation in amyotrophic lateral sclerosis. Ann Clin Transl Neurol.

[CR17] Filippini T, Tesauro M, Fiore M (2020). Environmental and occupational risk factors of amyotrophic lateral sclerosis: a population-based case-control study. Int J Environ Res Public Health.

[CR18] Ge CB, Friesen MC, Kromhout H (2018). Use and reliability of exposure assessment methods in occupational case-control studies in the general population: past, present, and future. Ann Work Expos Health.

[CR19] Goutman SA (2017). Diagnosis and clinical management of amyotrophic lateral sclerosis and other motor neuron disorders. Continuum (minneap Minn)..

[CR20] Goutman SA, Feldman EL (2020). Voicing the need for amyotrophic lateral sclerosis environmental research. JAMA Neurol.

[CR21] Goutman SA, Boss J, Patterson A, Mukherjee B, Batterman S, Feldman EL (2019). High plasma concentrations of organic pollutants negatively impact survival in amyotrophic lateral sclerosis. J Neurol Neurosurg Psychiatry.

[CR22] Gresham LS, Molgaard CA, Golbeck AL, Smith R (1986). Amyotrophic lateral sclerosis and occupational heavy metal exposure: a case-control study. Neuroepidemiology.

[CR23] Gunnarsson LG, Bodin L, Söderfeldt B, Axelson O (1992). A case-control study of motor neurone disease: its relation to heritability, and occupational exposures, particularly to solvents. Br J Ind Med.

[CR24] Jalilian H, Najafi K, Khosravi Y, Röösli M (2021). Amyotrophic lateral sclerosis, occupational exposure to extremely low frequency magnetic fields and electric shocks: a systematic review and meta-analysis. Rev Environ Health.

[CR25] Kamel F, Umbach DM, Munsat TL, Shefner JM, Hu H, Sandler DP (2002). Lead exposure and amyotrophic lateral sclerosis. Epidemiology.

[CR26] Kamel F, Umbach DM, Bedlack RS (2012). Pesticide exposure and amyotrophic lateral sclerosis. Neurotoxicology.

[CR27] Koeman T, Slottje P, Schouten LJ (2017). Occupational exposure and amyotrophic lateral sclerosis in a prospective cohort. Occup Environ Med.

[CR28] Malek AM, Barchowsky A, Bowser R (2014). Environmental and occupational risk factors for amyotrophic lateral sclerosis: a case-control study. Neurodegener Dis.

[CR29] McGuire V, Longstreth WT, Nelson LM (1997). Occupational exposures and amyotrophic lateral sclerosis. A population-based case-control study. Am J Epidemiol.

[CR30] Mixed Exposures Research Agenda (2004) A Report by the NORA Mixed Exposures Team

[CR31] Morahan JM, Pamphlett R (2006). Amyotrophic lateral sclerosis and exposure to environmental toxins: an Australian case-control study. Neuroepidemiology.

[CR32] OSHA (2021) Controlling Hazardous Fume and Gases during Welding. https://www.osha.gov/sites/default/files/publications/OSHA_FS-3647_Welding.pdf. . Accessed 13 May 2021

[CR33] Pamphlett R (2012). Exposure to environmental toxins and the risk of sporadic motor neuron disease: an expanded Australian case-control study. Eur J Neurol.

[CR34] Pamphlett R, Rikard-Bell A (2013). Different occupations associated with amyotrophic lateral sclerosis: is diesel exhaust the link?. PLoS ONE.

[CR35] Peters TL, Kamel F, Lundholm C (2017). Occupational exposures and the risk of amyotrophic lateral sclerosis. Occup Environ Med.

[CR36] Russ DE, Ho KY, Colt JS (2016). Computer-based coding of free-text job descriptions to efficiently identify occupations in epidemiological studies. Occup Environ Med.

[CR37] Strickland D, Smith SA, Dolliff G, Goldman L, Roelofs RI (1996). Amyotrophic lateral sclerosis and occupational history. A pilot case-control study. Arch Neurol.

[CR38] Su FC, Goutman SA, Chernyak S (2016). Association of environmental toxins with amyotrophic lateral sclerosis. JAMA Neurol.

[CR39] Teschke K, Olshan AF, Daniels JL (2002). Occupational exposure assessment in case-control studies: opportunities for improvement. Occup Environ Med.

[CR40] Visser AE, D'Ovidio F, Peters S (2019). Multicentre, population-based, case–control study of particulates, combustion products and amyotrophic lateral sclerosis risk. J Neurol Neurosurg Psychiatry.

[CR41] Wang MD, Little J, Gomes J, Cashman NR, Krewski D (2017). Identification of risk factors associated with onset and progression of amyotrophic lateral sclerosis using systematic review and meta-analysis. Neurotoxicology.

[CR42] Weisskopf MG, McCullough ML, Morozova N, Calle EE, Thun MJ, Ascherio A (2005). Prospective study of occupation and amyotrophic lateral sclerosis mortality. Am J Epidemiol.

[CR43] Yu Y, Su FC, Callaghan BC, Goutman SA, Batterman SA, Feldman EL (2014). Environmental risk factors and amyotrophic lateral sclerosis (ALS): a case-control study of ALS in Michigan. PLoS ONE.

