# Supplementary material for: Associations of self-reported occupational exposures and settings to ALS: a case–control study
Source: Int Arch Occup Environ Health. 2022 May 20;95(7):1567–86. doi: 10.1007/s00420-022-01874-4 (PMC9424174; doi:10.1007/s00420-022-01874-4)

# Title Page

Title: Associations of self-reported occupational exposures and settings to ALS: a case-control study

Authors: ^1,2^Stephen A Goutman, MD, MS (ORCID ID: 0000-0001-8780-6637), ^3^Jonathan Boss, ^4^Christopher Godwin, DDS, PhD, ^3^Bhramar Mukherjee, PhD, ^1,2^Eva L. Feldman, MD, PhD (ORCID ID: 0000-0002-9162-2694), ^4^Stuart A. Batterman, PhD

Affiliations:

^1^Department of Neurology, University of Michigan, Ann Arbor, MI
^2^NeuroNetwork for Emerging Therapies, University of Michigan, Ann Arbor, MI
^3^Department of Biostatistics, University of Michigan, Ann Arbor, MI

^4^Department of Environmental Health Sciences, University of Michigan, Ann Arbor, MI

# Supplemental Materials

# Supplemental Tables

## Table S1. Scores and questions

Questionnaire items used to develop occupational exposure scores for 9 exposure types. Applies to occupational exposures for jobs 1 to 4. Question paraphrases each item. Unnormalized scores are shown, and all scores were normalized (0 – 1) for statistical analysis. Notes: 1 – multiple questions about potential exposure to agent as well as contact by breathing, touching, or ingesting (swallowing); score is awarded if any question is answered “yes”. 2 - separate questions about each agent identified in question. Score is awarded if any question is answered “yes”. If indicated, scores are limited to maximum shown, e.g., VOC score does not exceed 12.

| **Type** |  | **Question** | **No. Vars.** | **Scoring** | **Note** | **Range of Scores** |
| --- | --- | --- | --- | --- | --- | --- |
|  |  |  |  |  |  |  |
| **Particulate Matter (PM)** | | |  |  |  |  |
|  | **Total exposure to PM at work** | | 18 | **sum of general + specific PM** |  | **0 - 12** |
|  | **Exposure to general sources of PM at work** | | **10** | **sum of below** |  | **0 - 6** |
|  |  | Exposed to silica powder, coal dust, rock dust, wood dust or other dusts? | 5 | any yes = 2 | 2 |  |
|  |  | Exposed to asbestos, fiberglass or other fibers? | 3 | any yes = 2 | 2 |  |
|  |  | Exposed to welding fumes or gas, or diesel smoke? | 2 | any yes = 2 | 2 |  |
|  | **Exposure to specific sources of PM exposure at work** | |  | **sum of below** |  | **0 - 6** |
|  |  | Exposed to asbestos, coal dust, diesel exhaust, fiberglass, rock dust, silica powder, talc, or welding fumes without PPE? | 8 | count of exposure types ("yes") of 1, 2, or more = 2, 4, 6, respectively | 2 |  |
|  |  |  |  |  |  |  |
| **Volatile Organic Compounds (VOCs)** | | |  |  |  |  |
|  | **Exposure to VOCs at work** | | **36** | **sum of below, maximum of 12** |  | **0 - 12** |
|  |  | Exposed to alcohols (non-beverage)? | 2 | any yes = 1 | 1 |  |
|  |  | Exposed to benzene? | 2 | any yes = 3 | 1 |  |
|  |  | Exposed to phenyl? | 2 | any yes = 2 | 1 |  |
|  |  | Exposed to chloroform? | 2 | any yes = 3 | 1 |  |
|  |  | Exposed to toluene? | 2 | any yes = 2 | 1 |  |
|  |  | Exposed to carbon tetrachloride? | 2 | any yes = 3 | 1 |  |
|  |  | Exposed to chloroprene? | 2 | any yes = 2 | 1 |  |
|  |  | Exposed to PCBs or PBBs | 3 | any yes = 3 | 2 |  |
|  |  | Exposed to oils and lubricants? | 1 | yes = 1 |  |  |
|  |  | Exposed to methylene chloride (e.g., paint stripper)? | 2 | any yes = 3 | 1 |  |
|  |  | Exposed to halothene (general anesthetics)? | 2 | any yes = 1 | 1 |  |
|  |  | Washing hands with solvents? | 1 | yes = 3 |  |  |
|  |  | Exposed to other chlorinated solvents (including chlorinated naphthalenes, dichlorobenzene, perchloroethylene, trichloroethylene, vinyl chloride? | 5 | any yes = 3 | 2 |  |
|  |  | Exposed to ethylene dibromide or ethylene dichloride? | 2 | any yes = 2 | 1 |  |
|  |  | Exposed to isocyanates? | 1 | any yes = 2 |  |  |
|  |  | Exposed to ketones, trinitrotoluene, other solvents? | 3 | any yes = 1 | 2 |  |
|  |  | Exposed to styrene, TDI or MDI? | 2 | any yes = 2 | 2 |  |
|  |  |  |  |  |  |  |
| **Pesticides** | |  |  |  |  |  |
|  |  | Exposed to pesticides or arsenic containing compounds? | 2 | if any yes = 3 | 1 | **0 - 3** |
|  |  |  |  |  |  |  |
| **Metals** |  |  |  |  |  |  |
|  | **Total exposure to metals at work** | | 17 | **sum of specific metals + welding fumes** |  | **0 – 18** |
|  | **Exposure to specific metals at work** | | **15** | **sum of below, maximum of 12** |  | **0 – 12** |
|  |  | Exposed to iron? | 1 | yes = 1 |  |  |
|  |  | Exposed to lead? | 2 | any yes = 5 | 1 |  |
|  |  | Exposed to mercury? | 2 | any yes = 5 | 1 |  |
|  |  | Exposed to cadmium? | 2 | any yes = 3 | 1 |  |
|  |  | Exposed to beryllium? | 2 | any yes = 3 | 1 |  |
|  |  | Exposed to nickel? | 2 | any yes = 3 | 1 |  |
|  |  | Exposed to aluminum? | 2 | any yes = 3 | 1 |  |
|  |  | Exposed to arsenic? | 2 | any yes = 3 | 1 |  |
|  | **Exposed to welding fumes?** | | **2** | **any yes = 6** | 1 | **0 - 6** |
|  |  |  |  |  |  |  |
| **Biologicals** | |  |  |  |  |  |
|  |  | Exposed to animal fibers (including wool)? | 1 | yes = 1 |  | 0 - 1 |
|  |  |  |  |  |  |  |
| **Combustion Products** | | |  |  |  |  |
|  |  | Exposed to combustion products, including diesel exhaust or smoke? | 2 | any yes = 2 |  | 0 - 4 |
|  |  |  |  |  |  |  |
| **Electromagnetic Radiation (EM, non-ionizing)** | | |  |  |  |  |
|  |  | Exposed to power lines, transformation stations or other EM? | 3 | any yes = 2 | 2 | 0 - 2 |
|  |  |  |  |  |  |  |
| **Radiation (ionizing)** | | |  |  |  |  |
|  |  | Exposed to X-rays, radiotherapy, radioactive iodine therapy, other radiation? | **6** | **any yes = 2** | **1** | **0 - 2** |
|  |  |  |  |  |  |  |
| **Corrosives** | |  |  |  |  |  |
|  |  | Exposed to chemicals or vapors that are acid, alkali, ammonia, phosgene, other? | **5** | any yes = 3, multiple yes = 5 | 2 | **0 - 5** |

## Table S2. Numbers of individuals assessed for participation by category and age group

ALS participants can elect to participate in our biorepository study but not return a survey (“ALS, consented and declined survey” category) or can participate in our biorepository study and return a survey (“ALS, consented and returned survey” category).

|  | **Age group (years)** | | | | | |
| --- | --- | --- | --- | --- | --- | --- |
| **Participant Category** | **20-30** | **30-40** | **40-50** | **50-60** | **60-70** | **>70** |
| **ALS, consented and returned survey** | 2 | 7 | 40 | 93 | 145 | 94 |
| **ALS, consented and declined survey** | 4 | 18 | 53 | 91 | 87 | 63 |
| **ALS, declined research visit** | 3 | 12 | 28 | 63 | 112 | 80 |
| **Controls, in recruitment database** | 842 | 221 | 261 | 287 | 253 | 71 |
| **Controls, able to be contacted** | ^a^84 | ^a^36 | 56 | 124 | 151 | 46 |
| **Controls, consented** | 4 | 5 | 28 | 79 | 110 | 46 |
| ^a^Fewer individuals contacted intentionally due to small ALS participant numbers in this age category | | | | | | |

## Table S3. Occupational Exposure Scores

For all scores, minimum value is 0 and maximum is 1. Median for all scores is 0.

N, number; SD, standard deviation; Q, quartile.

|  | **Occupational exposure score** | | | | | | | | | | | | |
| --- | --- | --- | --- | --- | --- | --- | --- | --- | --- | --- | --- | --- | --- |
|  | **ALS (N = 381)** | | | | | | **Control N = 272)** | | | | | |  |
| **Exposure** | **N** | **Mean** | **SD** | **Q75** | **Q90** | **Q95** | **N** | **Mean** | **SD** | **Q75** | **Q90** | **Q95** | **P-Value** |
| **Particulate Matter (PM)** | 381 | 0.13 | 0.22 | 0.17 | 0.44 | 0.61 | 272 | 0.07 | 0.16 | 0.08 | 0.25 | 0.42 | 0.00 |
| **Volatile Organic Compounds (VOCs)** | 381 | 0.14 | 0.21 | 0.22 | 0.40 | 0.61 | 272 | 0.10 | 0.17 | 0.13 | 0.33 | 0.42 | 0.01 |
| **Pesticides** | 373 | 0.09 | 0.24 | 0.00 | 0.47 | 0.67 | 269 | 0.06 | 0.19 | 0.00 | 0.25 | 0.50 | 0.09 |
| **Metals** | 381 | 0.11 | 0.21 | 0.17 | 0.40 | 0.58 | 272 | 0.05 | 0.14 | 0.00 | 0.20 | 0.35 | 0.00 |
| **Biological Exposures** | 376 | 0.01 | 0.08 | 0.00 | 0.00 | 0.00 | 270 | 0.01 | 0.10 | 0.00 | 0.00 | 0.00 | 0.72 |
| **Combustion and Diesel Exhaust** | 379 | 0.13 | 0.32 | 0.00 | 1.00 | 1.00 | 270 | 0.08 | 0.25 | 0.00 | 0.25 | 1.00 | 0.02 |
| **Electromagnetic Exposure** | 381 | 0.08 | 0.22 | 0.00 | 0.33 | 0.50 | 272 | 0.05 | 0.19 | 0.00 | 0.23 | 0.41 | 0.12 |
| **Radiation** | 381 | 0.06 | 0.19 | 0.00 | 0.25 | 0.50 | 272 | 0.05 | 0.18 | 0.00 | 0.23 | 0.5 | 0.76 |
| **Corrosives** | 378 | 0.07 | 0.18 | 0.00 | 0.30 | 0.50 | 271 | 0.06 | 0.16 | 0.00 | 0.25 | 0.45 | 0.66 |

## Table S4. Summary Occupational Exposure Scores by Sex

| **Exposure** | **Sex** | **N** | **Mean** | **SD** | **Min** | **Q25** | **Q50** | **Q75** | **Max** | **P-value** |
| --- | --- | --- | --- | --- | --- | --- | --- | --- | --- | --- |
| **Particulate Matter** | Female | 316 | 0.03 | 0.07 | 0 | 0 | 0 | 0 | 0.5 | <0.001 |
|  | Male | 337 | 0.18 | 0.25 | 0 | 0 | 0.09 | 0.33 | 1 |  |
| **Volatile Organic Compounds (VOCs)** | Female | 316 | 0.06 | 0.11 | 0 | 0 | 0 | 0.07 | 0.5 | <0.001 |
|  | Male | 337 | 0.18 | 0.24 | 0 | 0 | 0.08 | 0.25 | 1 |  |
| **Pesticides** | Female | 309 | 0.05 | 0.17 | 0 | 0 | 0 | 0 | 1 | <0.001 |
|  | Male | 333 | 0.11 | 0.26 | 0 | 0 | 0 | 0 | 1 |  |
| **Metals** | Female | 316 | 0.02 | 0.08 | 0 | 0 | 0 | 0 | 0.56 | <0.001 |
|  | Male | 337 | 0.15 | 0.23 | 0 | 0 | 0 | 0.25 | 1 |  |
| **Biological Exposures** | Female | 314 | 0.01 | 0.05 | 0 | 0 | 0 | 0 | 0.67 | 0.055 |
|  | Male | 332 | 0.02 | 0.12 | 0 | 0 | 0 | 0 | 1 |  |
| **Combustion and Diesel Exhaust** | Female | 316 | 0.04 | 0.18 | 0 | 0 | 0 | 0 | 1 | <0.001 |
|  | Male | 333 | 0.18 | 0.36 | 0 | 0 | 0 | 0 | 1 |  |
| **Electromagnetic Exposure** | Female | 316 | 0.01 | 0.07 | 0 | 0 | 0 | 0 | 0.75 | <0.001 |
|  | Male | 337 | 0.12 | 0.27 | 0 | 0 | 0 | 0 | 1 |  |
| **Radiation** | Female | 316 | 0.06 | 0.2 | 0 | 0 | 0 | 0 | 1 | 0.549 |
|  | Male | 337 | 0.05 | 0.17 | 0 | 0 | 0 | 0 | 1 |  |
| **Corrosives** | Female | 316 | 0.03 | 0.11 | 0 | 0 | 0 | 0 | 0.67 | <0.001 |
|  | Male | 333 | 0.09 | 0.21 | 0 | 0 | 0 | 0 | 1 |  |

N, number; SD, standard deviation; Min, minimum, Q25, first quartile; Q50, median; Q75, third quartile; Max, maximum

## Table S4a. Summary Occupational Exposure Scores by Sex and by ALS and Control Status

|  |  | **ALS** | | | | | | | | **Controls** | | | | | | | |
| --- | --- | --- | --- | --- | --- | --- | --- | --- | --- | --- | --- | --- | --- | --- | --- | --- | --- |
| **Exposure** | **Sex** | **N** | **Mean** | **SD** | **Min** | **Q25** | **Q50** | **Q75** | **Max** | **N** | **Mean** | **SD** | **Min** | **Q25** | **Q50** | **Q75** | **Max** |
| **Particulate Matter** | Female | 172 | 0.03 | 0.07 | 0 | 0 | 0 | 0 | 0.50 | 144 | 0.02 | 0.07 | 0 | 0 | 0 | 0 | 0.44 |
|  | Male | 209 | 0.22 | 0.26 | 0 | 0 | 0.12 | 0.34 | 1.00 | 128 | 0.13 | 0.20 | 0 | 0 | 0 | 0.17 | 1.00 |
| **Volatile Organic Compounds (VOCs)** | Female | 172 | 0.06 | 0.11 | 0 | 0 | 0 | 0.07 | 0.50 | 144 | 0.06 | 0.11 | 0 | 0 | 0 | 0.07 | 0.50 |
|  | Male | 209 | 0.20 | 0.25 | 0 | 0 | 0.12 | 0.33 | 1.00 | 128 | 0.14 | 0.22 | 0 | 0 | 0.02 | 0.22 | 1.00 |
| **Pesticides** | Female | 167 | 0.04 | 0.16 | 0 | 0 | 0 | 0 | 1.00 | 142 | 0.05 | 0.18 | 0 | 0 | 0 | 0 | 1.00 |
|  | Male | 206 | 0.13 | 0.28 | 0 | 0 | 0 | 0 | 1.00 | 127 | 0.07 | 0.21 | 0 | 0 | 0 | 0 | 1.00 |
| **Metals** | Female | 172 | 0.03 | 0.09 | 0 | 0 | 0 | 0 | 0.56 | 144 | 0.02 | 0.06 | 0 | 0 | 0 | 0 | 0.53 |
|  | Male | 209 | 0.19 | 0.25 | 0 | 0 | 0.06 | 0.33 | 1.00 | 128 | 0.09 | 0.19 | 0 | 0 | 0 | 0.11 | 1.00 |
| **Biological Exposures** | Female | 170 | 0.01 | 0.05 | 0 | 0 | 0 | 0 | 0.67 | 144 | 0.01 | 0.05 | 0 | 0 | 0 | 0 | 0.50 |
|  | Male | 206 | 0.02 | 0.10 | 0 | 0 | 0 | 0 | 1.00 | 126 | 0.02 | 0.14 | 0 | 0 | 0 | 0 | 1.00 |
| **Combustion and Diesel Exhaust** | Female | 172 | 0.05 | 0.19 | 0 | 0 | 0 | 0 | 1.00 | 144 | 0.03 | 0.15 | 0 | 0 | 0 | 0 | 1.00 |
|  | Male | 207 | 0.21 | 0.38 | 0 | 0 | 0 | 0.25 | 1.00 | 126 | 0.13 | 0.32 | 0 | 0 | 0 | 0 | 1.00 |
| **Electromagnetic Exposure** | Female | 172 | 0.01 | 0.06 | 0 | 0 | 0 | 0 | 0.50 | 144 | 0.01 | 0.08 | 0 | 0 | 0 | 0 | 0.75 |
|  | Male | 209 | 0.13 | 0.29 | 0 | 0 | 0 | 0 | 1.00 | 128 | 0.10 | 0.25 | 0 | 0 | 0 | 0 | 1.00 |
| **Radiation** | Female | 172 | 0.06 | 0.20 | 0 | 0 | 0 | 0 | 1.00 | 144 | 0.06 | 0.19 | 0 | 0 | 0 | 0 | 1.00 |
|  | Male | 209 | 0.05 | 0.18 | 0 | 0 | 0 | 0 | 1.00 | 128 | 0.05 | 0.17 | 0 | 0 | 0 | 0 | 1.00 |
| **Corrosives** | Female | 172 | 0.02 | 0.08 | 0 | 0 | 0 | 0 | 0.60 | 144 | 0.05 | 0.14 | 0 | 0 | 0 | 0 | 1.00 |
|  | Male | 206 | 0.11 | 0.22 | 0 | 0 | 0 | 0 | 1.00 | 127 | 0.07 | 0.19 | 0 | 0 | 0 | 0 | 1.00 |

N, number; SD, standard deviation; Min, minimum, Q25, first quartile; Q50, median; Q75, third quartile; Max, maximum

## Table S5. Summary Occupational Exposure Scores by Education

| **Exposure** | **Education** | **N** | **Mean** | **SD** | **Min** | **Q25** | **Q50** | **Q75** | **Max** | **P-value^1^** |
| --- | --- | --- | --- | --- | --- | --- | --- | --- | --- | --- |
| **Particulate Matter** | Bachelor's degree | 168 | 0.08 | 0.13 | 0 | 0 | 0 | 0.11 | 0.67 | <0.001 |
|  | Graduate | 149 | 0.05 | 0.11 | 0 | 0 | 0 | 0.04 | 0.67 |  |
|  | HS or Less | 129 | 0.17 | 0.27 | 0 | 0 | 0 | 0.28 | 1 |  |
|  | Some postsecondary | 200 | 0.14 | 0.23 | 0 | 0 | 0 | 0.17 | 1 |  |
|  | NA | 7 | 0.06 | 0.12 | 0 | 0 | 0 | 0.04 | 0.33 |  |
| **Volatile Organic Compounds (VOCs)** | Bachelor's degree | 168 | 0.09 | 0.15 | 0 | 0 | 0 | 0.14 | 1 | 0.013 |
|  | Graduate | 149 | 0.05 | 0.11 | 0 | 0 | 0 | 0.06 | 0.69 |  |
|  | HS or Less | 129 | 0.16 | 0.22 | 0 | 0 | 0.06 | 0.25 | 1 |  |
|  | Some postsecondary | 200 | 0.17 | 0.24 | 0 | 0 | 0.06 | 0.28 | 1 |  |
|  | NA | 7 | 0.08 | 0.09 | 0 | 0.02 | 0.08 | 0.1 | 0.25 |  |
| **Pesticides** | Bachelor's degree | 165 | 0.05 | 0.14 | 0 | 0 | 0 | 0 | 0.67 | 0.026 |
|  | Graduate | 149 | 0.05 | 0.16 | 0 | 0 | 0 | 0 | 1 |  |
|  | HS or Less | 126 | 0.12 | 0.28 | 0 | 0 | 0 | 0 | 1 |  |
|  | Some postsecondary | 195 | 0.1 | 0.27 | 0 | 0 | 0 | 0 | 1 |  |
|  | NA | 7 | 0 | 0 | 0 | 0 | 0 | 0 | 0 |  |
| **Metals** | Bachelor's degree | 168 | 0.06 | 0.14 | 0 | 0 | 0 | 0.04 | 0.78 | 0.039 |
|  | Graduate | 149 | 0.04 | 0.11 | 0 | 0 | 0 | 0 | 0.64 |  |
|  | HS or Less | 129 | 0.12 | 0.21 | 0 | 0 | 0 | 0.19 | 1 |  |
|  | Some postsecondary | 200 | 0.13 | 0.24 | 0 | 0 | 0 | 0.19 | 1 |  |
|  | NA | 7 | 0.1 | 0.17 | 0 | 0 | 0 | 0.14 | 0.4 |  |
| **Biological Exposures** | Bachelor's degree | 168 | 0.01 | 0.09 | 0 | 0 | 0 | 0 | 0.67 | 0.39 |
|  | Graduate | 146 | 0 | 0.03 | 0 | 0 | 0 | 0 | 0.25 |  |
|  | HS or Less | 128 | 0.02 | 0.13 | 0 | 0 | 0 | 0 | 1 |  |
|  | Some postsecondary | 197 | 0.02 | 0.09 | 0 | 0 | 0 | 0 | 1 |  |
|  | NA | 7 | 0 | 0 | 0 | 0 | 0 | 0 | 0 |  |
| **Combustion and Diesel Exhaust** | Bachelor's degree | 168 | 0.11 | 0.28 | 0 | 0 | 0 | 0 | 1 | <0.001 |
|  | Graduate | 148 | 0.06 | 0.22 | 0 | 0 | 0 | 0 | 1 |  |
|  | HS or Less | 128 | 0.19 | 0.37 | 0 | 0 | 0 | 0 | 1 |  |
|  | Some postsecondary | 198 | 0.11 | 0.3 | 0 | 0 | 0 | 0 | 1 |  |
|  | NA | 7 | 0.04 | 0.09 | 0 | 0 | 0 | 0 | 0.25 |  |
| **Electromagnetic Exposure** | Bachelor's degree | 168 | 0.07 | 0.21 | 0 | 0 | 0 | 0 | 1 | 0.012 |
|  | Graduate | 149 | 0.01 | 0.05 | 0 | 0 | 0 | 0 | 0.25 |  |
|  | HS or Less | 129 | 0.11 | 0.27 | 0 | 0 | 0 | 0 | 1 |  |
|  | Some postsecondary | 200 | 0.09 | 0.23 | 0 | 0 | 0 | 0 | 1 |  |
|  | NA | 7 | 0 | 0 | 0 | 0 | 0 | 0 | 0 |  |
| **Radiation** | Bachelor's degree | 168 | 0.06 | 0.18 | 0 | 0 | 0 | 0 | 1 | 0.52 |
|  | Graduate | 149 | 0.02 | 0.09 | 0 | 0 | 0 | 0 | 0.75 |  |
|  | HS or Less | 129 | 0.05 | 0.18 | 0 | 0 | 0 | 0 | 1 |  |
|  | Some postsecondary | 200 | 0.08 | 0.24 | 0 | 0 | 0 | 0 | 1 |  |
|  | NA | 7 | 0.04 | 0.09 | 0 | 0 | 0 | 0 | 0.25 |  |
| **Corrosives** | Bachelor's degree | 168 | 0.06 | 0.15 | 0 | 0 | 0 | 0 | 0.65 | 0.11 |
|  | Graduate | 149 | 0.03 | 0.12 | 0 | 0 | 0 | 0 | 0.87 |  |
|  | HS or Less | 127 | 0.09 | 0.22 | 0 | 0 | 0 | 0 | 1 |  |
|  | Some postsecondary | 198 | 0.07 | 0.19 | 0 | 0 | 0 | 0 | 1 |  |
|  | NA | 7 | 0.02 | 0.06 | 0 | 0 | 0 | 0 | 0.15 |  |

^1^Test for differences for participants with a high school or less education compared to those with some postsecondary education, college degree, or graduate education

N, number; SD, standard deviation; Min, minimum, Q25, first quartile; Q50, median; Q75, third quartile; Max, maximum

## Table S5a. Summary Occupational Exposure Scores by Education and by ALS and Control Status

|  |  | **ALS** | | | | | | | | **Control** | | | | | | | |
| --- | --- | --- | --- | --- | --- | --- | --- | --- | --- | --- | --- | --- | --- | --- | --- | --- | --- |
| **Exposure** | **Education** | **N** | **Mean** | **SD** | **Min** | **Q25** | **Q50** | **Q75** | **Max** | **N** | **Mean** | **SD** | **Min** | **Q25** | **Q50** | **Q75** | **Max** |
| **Particulate Matter** | Bachelor's degree | 87 | 0.08 | 0.14 | 0 | 0 | 0 | 0.10 | 0.67 | 81 | 0.07 | 0.13 | 0 | 0 | 0 | 0.11 | 0.54 |
|  | Graduate | 61 | 0.07 | 0.14 | 0 | 0 | 0 | 0.08 | 0.67 | 88 | 0.04 | 0.09 | 0 | 0 | 0 | 0 | 0.55 |
|  | HS or Less | 106 | 0.17 | 0.27 | 0 | 0 | 0 | 0.25 | 1.00 | 23 | 0.16 | 0.26 | 0 | 0 | 0 | 0.33 | 1.00 |
|  | Some postsecondary | 123 | 0.17 | 0.25 | 0 | 0 | 0.04 | 0.33 | 1.00 | 77 | 0.09 | 0.19 | 0 | 0 | 0 | 0.09 | 1.00 |
|  | NA | 4 | 0.08 | 0.17 | 0 | 0 | 0 | 0.08 | 0.33 | 3 | 0.03 | 0.05 | 0 | 0 | 0 | 0.04 | 0.09 |
| **Volatile Organic Compounds (VOCs)** | Bachelor's degree | 87 | 0.10 | 0.17 | 0 | 0 | 0 | 0.16 | 1.00 | 81 | 0.07 | 0.14 | 0 | 0 | 0 | 0.10 | 0.69 |
|  | Graduate | 61 | 0.05 | 0.10 | 0 | 0 | 0 | 0.04 | 0.46 | 88 | 0.06 | 0.12 | 0 | 0 | 0 | 0.07 | 0.69 |
|  | HS or Less | 106 | 0.15 | 0.22 | 0 | 0 | 0.05 | 0.25 | 1.00 | 23 | 0.18 | 0.25 | 0 | 0 | 0.06 | 0.29 | 1.00 |
|  | Some postsecondary | 123 | 0.19 | 0.26 | 0 | 0 | 0.08 | 0.33 | 1.00 | 77 | 0.14 | 0.21 | 0 | 0 | 0.04 | 0.21 | 1.00 |
|  | NA | 4 | 0.11 | 0.10 | 0 | 0.06 | 0.10 | 0.16 | 0.25 | 3 | 0.04 | 0.04 | 0 | 0.02 | 0.04 | 0.06 | 0.08 |
| **Pesticides** | Bachelor's degree | 86 | 0.05 | 0.13 | 0 | 0 | 0 | 0 | 0.50 | 79 | 0.06 | 0.15 | 0 | 0 | 0 | 0 | 0.67 |
|  | Graduate | 61 | 0.07 | 0.18 | 0 | 0 | 0 | 0 | 0.67 | 88 | 0.03 | 0.14 | 0 | 0 | 0 | 0 | 1.00 |
|  | HS or Less | 103 | 0.12 | 0.28 | 0 | 0 | 0 | 0 | 1.00 | 23 | 0.13 | 0.31 | 0 | 0 | 0 | 0 | 1.00 |
|  | Some postsecondary | 119 | 0.11 | 0.28 | 0 | 0 | 0 | 0 | 1.00 | 76 | 0.08 | 0.24 | 0 | 0 | 0 | 0 | 1.00 |
|  | NA | 4 | 0 | 0 | 0 | 0 | 0 | 0 | 0 | 3 | 0 | 0 | 0 | 0 | 0 | 0 | 0 |
| **Metals** | Bachelor's degree | 87 | 0.06 | 0.14 | 0 | 0 | 0 | 0.04 | 0.78 | 81 | 0.06 | 0.13 | 0 | 0 | 0 | 0.01 | 0.76 |
|  | Graduate | 61 | 0.06 | 0.13 | 0 | 0 | 0 | 0.07 | 0.64 | 88 | 0.03 | 0.09 | 0 | 0 | 0 | 0 | 0.51 |
|  | HS or Less | 106 | 0.12 | 0.21 | 0 | 0 | 0 | 0.22 | 1.00 | 23 | 0.10 | 0.19 | 0 | 0 | 0 | 0 | 0.61 |
|  | Some postsecondary | 123 | 0.17 | 0.26 | 0 | 0 | 0 | 0.28 | 1.00 | 77 | 0.06 | 0.18 | 0 | 0 | 0 | 0 | 1.00 |
|  | NA | 4 | 0.10 | 0.20 | 0 | 0 | 0 | 0.10 | 0.40 | 3 | 0.09 | 0.16 | 0 | 0 | 0 | 0.14 | 0.28 |
| **Biological Exposures** | Bachelor's degree | 87 | 0.02 | 0.10 | 0 | 0 | 0 | 0 | 0.67 | 81 | 0.01 | 0.06 | 0 | 0 | 0 | 0 | 0.50 |
|  | Graduate | 59 | 0.00 | 0.03 | 0 | 0 | 0 | 0 | 0.25 | 87 | 0.00 | 0.03 | 0 | 0 | 0 | 0 | 0.25 |
|  | HS or Less | 105 | 0.01 | 0.11 | 0 | 0 | 0 | 0 | 1.00 | 23 | 0.04 | 0.21 | 0 | 0 | 0 | 0 | 1.00 |
|  | Some postsecondary | 121 | 0.01 | 0.06 | 0 | 0 | 0 | 0 | 0.50 | 76 | 0.03 | 0.13 | 0 | 0 | 0 | 0 | 1.00 |
|  | NA | 4 | 0 | 0 | 0 | 0 | 0 | 0 | 0 | 3 | 0 | 0 | 0 | 0 | 0 | 0 | 0 |
| **Combustion and Diesel Exhaust** | Bachelor's degree | 87 | 0.09 | 0.27 | 0 | 0 | 0 | 0 | 1.00 | 81 | 0.12 | 0.29 | 0 | 0 | 0 | 0 | 1.00 |
|  | Graduate | 61 | 0.08 | 0.25 | 0 | 0 | 0 | 0 | 1.00 | 87 | 0.04 | 0.19 | 0 | 0 | 0 | 0 | 1.00 |
|  | HS or Less | 105 | 0.21 | 0.38 | 0 | 0 | 0 | 0.25 | 1.00 | 23 | 0.11 | 0.30 | 0 | 0 | 0 | 0 | 1.00 |
|  | Some postsecondary | 122 | 0.13 | 0.32 | 0 | 0 | 0 | 0 | 1.00 | 76 | 0.07 | 0.25 | 0 | 0 | 0 | 0 | 1.00 |
|  | NA | 4 | 0.06 | 0.12 | 0 | 0 | 0 | 0.06 | 0.25 | 3 | 0 | 0 | 0 | 0 | 0 | 0 | 0 |
| **Electromagnetic Exposure** | Bachelor's degree | 87 | 0.07 | 0.19 | 0 | 0 | 0 | 0 | 1.00 | 81 | 0.08 | 0.22 | 0 | 0 | 0 | 0 | 1.00 |
|  | Graduate | 61 | 0.00 | 0.03 | 0 | 0 | 0 | 0 | 0.25 | 88 | 0.01 | 0.05 | 0 | 0 | 0 | 0 | 0.25 |
|  | HS or Less | 106 | 0.12 | 0.29 | 0 | 0 | 0 | 0 | 1.00 | 23 | 0.06 | 0.22 | 0 | 0 | 0 | 0 | 1.00 |
|  | Some postsecondary | 123 | 0.09 | 0.24 | 0 | 0 | 0 | 0 | 1.00 | 77 | 0.07 | 0.23 | 0 | 0 | 0 | 0 | 1.00 |
|  | NA | 4 | 0 | 0 | 0 | 0 | 0 | 0 | 0 | 3 | 0 | 0 | 0 | 0 | 0 | 0 | 0 |
| **Radiation** | Bachelor's degree | 87 | 0.05 | 0.16 | 0 | 0 | 0 | 0 | 1.00 | 81 | 0.08 | 0.20 | 0 | 0 | 0 | 0 | 1.00 |
|  | Graduate | 61 | 0.02 | 0.09 | 0 | 0 | 0 | 0 | 0.50 | 88 | 0.02 | 0.09 | 0 | 0 | 0 | 0 | 0.75 |
|  | HS or Less | 106 | 0.05 | 0.19 | 0 | 0 | 0 | 0 | 1.00 | 23 | 0.04 | 0.12 | 0 | 0 | 0 | 0 | 0.50 |
|  | Some postsecondary | 123 | 0.09 | 0.24 | 0 | 0 | 0 | 0 | 1.00 | 77 | 0.07 | 0.24 | 0 | 0 | 0 | 0 | 1.00 |
|  | NA | 4 | 0 | 0 | 0 | 0 | 0 | 0 | 0 | 3 | 0.08 | 0.14 | 0 | 0 | 0 | 0.12 | 0.25 |
| **Corrosives** | Bachelor's degree | 87 | 0.05 | 0.14 | 0 | 0 | 0 | 0 | 0.65 | 81 | 0.07 | 0.15 | 0 | 0 | 0 | 0 | 0.65 |
|  | Graduate | 61 | 0.03 | 0.09 | 0 | 0 | 0 | 0 | 0.40 | 88 | 0.04 | 0.14 | 0 | 0 | 0 | 0 | 0.87 |
|  | HS or Less | 104 | 0.08 | 0.22 | 0 | 0 | 0 | 0 | 1.00 | 23 | 0.09 | 0.22 | 0 | 0 | 0 | 0 | 0.67 |
|  | Some postsecondary | 122 | 0.08 | 0.20 | 0 | 0 | 0 | 0 | 1.00 | 76 | 0.06 | 0.17 | 0 | 0 | 0 | 0 | 1.00 |
|  | NA | 4 | 0 | 0 | 0 | 0 | 0 | 0 | 0 | 3 | 0.05 | 0.09 | 0 | 0 | 0 | 0.07 | 0.15 |

N, number; SD, standard deviation; Min, minimum, Q25, first quartile; Q50, median; Q75, third quartile; Max, maximum

## Table S6. ALS and Control Logistic Regression Models

Single exposure score logistic regression and multivariable logistic regression models where the outcome is ALS/control status, the variables of interest are the occupational exposure scores, and the covariates are age, sex, and military service. The occupational exposure scores are not weighted by duration of occupation. AEN, adaptive elastic net; OR, odds ratio; CI, confidence interval.

|  | **Occupational exposure scores** | | | | | | |
| --- | --- | --- | --- | --- | --- | --- | --- |
|  | **Univariate Model** | | | **Multivariable Model** | | | **AEN** |
| **Exposure Score** | **OR** | **95% CI** | **P-Value** | **OR** | **95% CI** | **P-Value** | **OR** |
| **Particulate Matter (PM)** | 1.41 | 1.15-1.73 | 0.001 | 1.18 | 0.84-1.66 | 0.349 | 1.095 |
| **Volatile Organic Compounds (VOCs)** | 1.22 | 1.02-1.46 | 0.029 | 1.02 | 0.78-1.32 | 0.909 | 1.034 |
| **Pesticides** | 1.13 | 0.95-1.34 | 0.161 | 1.03 | 0.85-1.27 | 0.740 | 1.000 |
| **Metals** | 1.45 | 1.18-1.79 | <0.001 | 1.56 | 1.10-2.22 | 0.013 | 1.136 |
| **Biologicals** | 0.97 | 0.83-1.14 | 0.706 | 0.92 | 0.77-1.09 | 0.333 | 0.979 |
| **Combustion and Diesel Exhaust** | 1.19 | 1.00-1.42 | 0.046 | 1.05 | 0.86-1.30 | 0.622 | 1.042 |
| **Electromagnetic Radiation** | 1.09 | 0.92-1.30 | 0.322 | 0.91 | 0.73-1.13 | 0.394 | 1.000 |
| **Radiation** | 1.04 | 0.89-1.22 | 0.640 | 0.93 | 0.77-1.12 | 0.437 | 1.000 |
| **Corrosives** | 1.03 | 0.88-1.21 | 0.726 | 0.82 | 0.66-1.01 | 0.059 | 0.967 |

## Table S7. Occupational Metals Associated with ALS Risk

Single exposure logistic regression models where the outcome is ALS/control status, the variables of interest

are indicators of ever having an occupational exposure, and the covariates are age, sex, and military service.

Interpretation of odds ratio is comparing a subject with no exposure to a subject with exposure at at least one job.

N, number; OR, odds ratio; LCL, lower confidence limit; UCL, upper confidence limit.

| **Exposure** | **N (Exposed)** | **OR** | **95% LCL** | **95% UCL** | **P-Value** |
| --- | --- | --- | --- | --- | --- |
| Iron | 75 | 2.25 | 1.25 | 4.02 | 0.006 |
| Lead | 98 | 1.1 | 0.69 | 1.74 | 0.686 |
| Mercury | 54 | 1.85 | 0.99 | 3.46 | 0.054 |
| Cadmium | 24 | 1.55 | 0.62 | 3.84 | 0.348 |
| Beryllium | 13 | 3.6 | 0.78 | 16.61 | 0.1 |
| Nickel | 45 | 1.35 | 0.7 | 2.6 | 0.367 |
| Aluminum | 49 | 1.69 | 0.88 | 3.27 | 0.115 |
| Arsenic | 17 | 3.08 | 0.87 | 10.94 | 0.082 |
| Welding Fumes | 128 | 1.97 | 1.25 | 3.11 | 0.003 |

## Table S8. SOC codes and aggregations

|  | **new aggregated code** | member SOC |  |  |  |  |  |  |  |  |  |  |  |  |  |  |  |
| --- | --- | --- | --- | --- | --- | --- | --- | --- | --- | --- | --- | --- | --- | --- | --- | --- | --- |
|  |  | **Paired SOC Codes and Descriptors** | | | | | | | | | | | | | | | |
| **Descriptor** | **Total** | **1** | **2** | **3** | **4** | **5** | **6** | **7** | **8** | **9** | **10** | **11** | **12** | **13** | **14** | **15** | **16** |
| SOC | **11-1011-AG02** | 11-1011 | 11-1021 |  |  |  |  |  |  |  |  |  |  |  |  |  |  |
| Short title | **Executive** | Executive | Manager, general |  |  |  |  |  |  |  |  |  |  |  |  |  |  |
| No. Persons | 50 | 16 | 34 |  |  |  |  |  |  |  |  |  |  |  |  |  |  |
| SOC | **11-2021-AG12** | 11-2021 | 11-2022 | 11-2031 | 11-3011 | 11-3021 | 11-3031 | 11-3061 | 11-3071 | 11-3111 | 11-3121 | 13-1071 | 13-1075 |  |  |  |  |
| Short title | **Manager, business** | Administrator, marketing | Manager, sales | Director, financial | Manager, services | Manager, IT | Manager, financial | Manager, purchasing | Manager, logistics | Administrator, financial | Director, HR | Manager, staffing | Manager, HR |  |  |  |  |
| No. Persons | 83 | 6 | 22 | 1 | 9 | 7 | 17 | 7 | 7 | 2 | 2 | 2 | 1 |  |  |  |  |
| SOC | **11-3051-AG02** | 11-3051 | 51-9061 |  |  |  |  |  |  |  |  |  |  |  |  |  |  |
| Short title | **Manager, production** | Manager, manufacturing | Inspector, production |  |  |  |  |  |  |  |  |  |  |  |  |  |  |
| No. Persons | 16 | 10 | 6 |  |  |  |  |  |  |  |  |  |  |  |  |  |  |
| SOC | **11-3131-AG06** | 11-3131 | 11-9032 | 11-9033 | 11-9039 | 11-9081 | 13-1151 |  |  |  |  |  |  |  |  |  |  |
| Short title | **Administrator, education** | Manager, education | Administrator, elementary education | Administrator, post secondary education, | Administrator, other education | Administrator, education | Trainer, computer |  |  |  |  |  |  |  |  |  |  |
| No. Persons | 25 | 1 | 8 | 11 | 2 | 2 | 1 |  |  |  |  |  |  |  |  |  |  |
| SOC | **11-9013-AG02** | 11-9013 | 25-9021 |  |  |  |  |  |  |  |  |  |  |  |  |  |  |
| Short title | **Manager, agriculture** | Managers, farming | Advisor, agricultural |  |  |  |  |  |  |  |  |  |  |  |  |  |  |
| No. Persons | 11 | 10 | 1 |  |  |  |  |  |  |  |  |  |  |  |  |  |  |
| SOC | **11-9021-AG13** | 11-9021 | 11-9141 | 13-1051 | 13-2021 | 17-1022 | 17-2021 | 17-2051 | 17-2081 | 17-2111 | 17-3022 | 17-3031 | 47-1011 | 47-4011 |  |  |  |
| Short title | **Manager, construction** | Manager, construction | Manager, property | Estimator, construction | Appraiser, property | Surveyor | Engineer, agriculture | Engineer, civil | Engineer, environmental | Engineer, safety | Technician, civil | Technician, surveyor | Supervisor, construction trades | Inspector, building |  |  |  |
| No. Persons | 27 | 2 | 6 | 2 | 1 | 1 | 1 | 5 | 1 | 1 | 1 | 2 | 3 | 1 |  |  |  |
| SOC | **11-9041-AG04** | 11-9041 | 11-9111 | 11-9121 | 11-9199 |  |  |  |  |  |  |  |  |  |  |  |  |
| Short title | **Manager, other professional** | Manager, engineering | Manager, medical services | Manager, research | Manager, professional |  |  |  |  |  |  |  |  |  |  |  |  |
| No. Persons | 37 | 8 | 9 | 3 | 17 |  |  |  |  |  |  |  |  |  |  |  |  |
| SOC | **11-9051** | 11-9051 |  |  |  |  |  |  |  |  |  |  |  |  |  |  |  |
| Short title | **Manager, food services** | Manager, food service |  |  |  |  |  |  |  |  |  |  |  |  |  |  |  |
| No. Persons | 8 | 8 |  |  |  |  |  |  |  |  |  |  |  |  |  |  |  |
| SOC | **11-9151-AG11** | 11-9151 | 21-1012 | 21-1015 | 21-1021 | 21-1022 | 21-1023 | 21-1091 | 21-1092 | 21-1093 | 21-2011 | 33-3012 |  |  |  |  |  |
| Short title | **Social worker** | Director, social services | Counselor, career | Counselor, rehab | Social worker, youth | Social worker, hospital | Social worker, mental health | Educator, community | Officer, parole | Assistant, family service | Clergy | Officer, correctional |  |  |  |  |  |
| No. Persons | 41 | 3 | 3 | 1 | 10 | 3 | 2 | 1 | 2 | 1 | 14 | 1 |  |  |  |  |  |
| SOC | **13-1022-AG02** | 13-1022 | 13-1023 |  |  |  |  |  |  |  |  |  |  |  |  |  |  |
| Short title | **Agent, buyer** | Buyer, wholesale and retail | Agent, purchasing |  |  |  |  |  |  |  |  |  |  |  |  |  |  |
| No. Persons | 5 | 3 | 2 |  |  |  |  |  |  |  |  |  |  |  |  |  |  |
| SOC | **13-1031-AG08** | 13-1031 | 43-3011 | 43-9021 | 43-9022 | 43-9041 | 43-9061 | 43-9071 | 43-9199 |  |  |  |  |  |  |  |  |
| Short title | **Clerk, office** | Adjuster, claims | Agent, account collection | Clerk, data | Clerk, typist | Clerk, insurance | Clerk, General | Operator, office machines | Clerk, office |  |  |  |  |  |  |  |  |
| No. Persons | 61 | 1 | 1 | 5 | 5 | 1 | 31 | 3 | 14 |  |  |  |  |  |  |  |  |
| SOC | **13-1041-AG02** | 13-1041 | 29-9011 |  |  |  |  |  |  |  |  |  |  |  |  |  |  |
| Short title | **Inspector, compliance, hygiene** | Officer, compliance | Hygienist, environmental and occupational |  |  |  |  |  |  |  |  |  |  |  |  |  |  |
| No. Persons | 5 | 4 | 1 |  |  |  |  |  |  |  |  |  |  |  |  |  |  |
| SOC | **13-1081-AG16** | 13-1081 | 13-1111 | 13-1131 | 13-1141 | 13-1161 | 13-1199 | 13-2011 | 13-2031 | 13-2041 | 13-2051 | 13-2052 | 13-2071 | 13-2072 | 13-2081 | 13-2082 | 13-2099 |
| Short title | **Analyst, technical** | Analyst, logistics | Analyst, business | Fundraiser | Analyst, Compensation | Analyst, marketing | Operations, business | Accountant | Analyst, budgets | Analyst, credit | Analyst, financial corporate | Analyst, financial individual | Counselors, credit | Officer, loans | Officers, IRS | Analysis, tax | Specialists, financial |
| No. Persons | 78 | 1 | 16 | 2 | 2 | 1 | 1 | 27 | 1 | 1 | 9 | 4 | 1 | 4 | 3 | 3 | 2 |
| SOC | **15-1121-AG10** | 15-1121 | 15-1131 | 15-1132 | 15-1133 | 15-1134 | 15-1141 | 15-1142 | 15-1143 | 15-1151 | 15-1199 |  |  |  |  |  |  |
| Short title | **Engineer, computer** | Analysts, IT | Programmer | Engineer, software | Programmer, systems | Programmer, web | Administrator, IT security | Administrator, IT network | Engineer, computer networks | Computer support specialist | Computer technician, other |  |  |  |  |  |  |
| No. Persons | 46 | 4 | 10 | 5 | 4 | 1 | 2 | 3 | 5 | 6 | 6 |  |  |  |  |  |  |
| SOC | **15-2031-AG04** | 15-2031 | 15-2041 | 17-2112 | 19-3011 |  |  |  |  |  |  |  |  |  |  |  |  |
| Short title | **Engineer, industrial** | Analyst, OR | Analyst, statistician | Engineer, industrial | Analyst, economic |  |  |  |  |  |  |  |  |  |  |  |  |
| No. Persons | 23 | 2 | 5 | 15 | 1 |  |  |  |  |  |  |  |  |  |  |  |  |
| SOC | **17-2011-AG07** | 17-2011 | 17-2041 | 17-2131 | 17-2141 | 17-2199 | 17-3027 | 17-3029 |  |  |  |  |  |  |  |  |  |
| Short title | **Engineer, mechanical** | Engineer, aerospace | Engineer, chemical | Engineer, mechanical | Engineer, mechanical | Engineer, other | Technician, mechanical | Technician, other |  |  |  |  |  |  |  |  |  |
| No. Persons | 45 | 5 | 1 | 1 | 14 | 14 | 1 | 9 |  |  |  |  |  |  |  |  |  |
| SOC | **17-2071-AG04** | 17-2071 | 17-2072 | 17-3023 | 53-6041 |  |  |  |  |  |  |  |  |  |  |  |  |
| Short title | **Engineer, electrical** | Engineer, electrical | Engineer, electronic | Technician, electrical | Technician, traffic controller |  |  |  |  |  |  |  |  |  |  |  |  |
| No. Persons | 30 | 6 | 7 | 16 | 1 |  |  |  |  |  |  |  |  |  |  |  |  |
| SOC | **17-3011-AG02** | 17-3011 | 17-3013 |  |  |  |  |  |  |  |  |  |  |  |  |  |  |
| Short title | **Drafter** | Drafters, architect | Drafters, automotive and machine |  |  |  |  |  |  |  |  |  |  |  |  |  |  |
| No. Persons | 12 | 6 | 6 |  |  |  |  |  |  |  |  |  |  |  |  |  |  |
| SOC | **19-1021-AG06** | 19-1021 | 19-1029 | 19-1032 | 19-1042 | 19-2012 | 19-2042 |  |  |  |  |  |  |  |  |  |  |
| Short title | **Researcher, scientist** | Scientist, biochemistry | Scientists, other | Scientist, ecologist | Scientist, Cancer | Scientist, dynamicist | Scientist, geochemist |  |  |  |  |  |  |  |  |  |  |
| No. Persons | 9 | 2 | 1 | 1 | 2 | 2 | 1 |  |  |  |  |  |  |  |  |  |  |
| SOC | **19-3031-AG03** | 19-3031 | 19-3032 | 19-3041 |  |  |  |  |  |  |  |  |  |  |  |  |  |
| Short title | **Psychologist** | Psychologist, clinical | Psychologists, engineering | Sociologist |  |  |  |  |  |  |  |  |  |  |  |  |  |
| No. Persons | 9 | 2 | 2 | 5 |  |  |  |  |  |  |  |  |  |  |  |  |  |
| SOC | **19-3093-AG05** | 19-3093 | 19-3099 | 27-3041 | 27-3042 | 27-3043 |  |  |  |  |  |  |  |  |  |  |  |
| Short title | **Writer** | Historian | Demographer | Writer, editor | Writer, technical | Writer, playwright |  |  |  |  |  |  |  |  |  |  |  |
| No. Persons | 13 | 1 | 1 | 1 | 5 | 5 |  |  |  |  |  |  |  |  |  |  |  |
| SOC | **19-4021-AG03** | 19-4021 | 19-4031 | 19-4099 |  |  |  |  |  |  |  |  |  |  |  |  |  |
| Short title | **Technican, laboratory** | Technician, biology | Technician, chemical | Technician, life science |  |  |  |  |  |  |  |  |  |  |  |  |  |
| No. Persons | 22 | 2 | 1 | 19 |  |  |  |  |  |  |  |  |  |  |  |  |  |
| SOC | **19-4061-AG12** | 19-4061 | 23-1012 | 23-2011 | 23-2091 | 25-4013 | 25-4021 | 29-2071 | 31-9094 | 43-6011 | 43-6012 | 43-6013 | 43-6014 |  |  |  |  |
| Short title | **Clerk, clerical** | Assistant, clerical | Clerk, legal | Legal, paralegal | Reporter, court | Conservator | Librarian | Technician, medical records | Transcriber, medical | Secretary, executive | Secretary, legal | Secretary, medical | Secretary, other |  |  |  |  |
| No. Persons | 110 | 10 | 1 | 4 | 2 | 1 | 1 | 1 | 2 | 8 | 5 | 2 | 73 |  |  |  |  |
| SOC | **23-1011-AG04** | 23-1011 | 23-1021 | 23-1022 | 23-1023 |  |  |  |  |  |  |  |  |  |  |  |  |
| Short title | **Judge or attorney** | Attorney | Judge | Arbitrator | Judge |  |  |  |  |  |  |  |  |  |  |  |  |
| No. Persons | 15 | 11 | 2 | 1 | 1 |  |  |  |  |  |  |  |  |  |  |  |  |
| SOC | **25-1021-AG08** | 25-1021 | 25-1022 | 25-1032 | 25-1041 | 25-1113 | 25-1123 | 25-1199 | 25-9031 |  |  |  |  |  |  |  |  |
| Short title | **Professor** | Professor, IT | Professor, math | Professor, English | Professor, Agriculture | Professor, Social work | Professor, English | Professor, misc | Education, curriculum |  |  |  |  |  |  |  |  |
| No. Persons | 17 | 1 | 2 | 2 | 1 | 1 | 4 | 2 | 4 |  |  |  |  |  |  |  |  |
| SOC | **25-1191-AG09** | 25-1191 | 25-2011 | 25-2021 | 25-2022 | 25-2031 | 25-2052 | 25-2054 | 25-3099 | 25-9041 |  |  |  |  |  |  |  |
| Short title | **Teacher, academic** | Teacher, GSI | Teacher, preschool | Teacher, elementary | Teacher, middle school | Teacher, high school | Teacher, special ed | Teacher, special ed, high school | Teacher, substitute | Aides, instruction |  |  |  |  |  |  |  |
| No. Persons | 99 | 6 | 3 | 31 | 5 | 13 | 1 | 4 | 21 | 15 |  |  |  |  |  |  |  |
| SOC | **25-1193-AG06** | 25-1193 | 25-3021 | 27-2021 | 27-2022 | 27-2031 | 39-9031 |  |  |  |  |  |  |  |  |  |  |
| Short title | **Teacher, sports** | Teacher, PE | Teacher, PE extra cur | Athlete, various | Coach, sports | Dancer | Instructor, fitness |  |  |  |  |  |  |  |  |  |  |
| No. Persons | 12 | 2 | 1 | 2 | 5 | 1 | 1 |  |  |  |  |  |  |  |  |  |  |
| SOC | **25-1194-AG02** | 25-1194 | 39-5012 |  |  |  |  |  |  |  |  |  |  |  |  |  |  |
| Short title | **Cosmetologist** | Instructor, cosmetology | Cosmetologist |  |  |  |  |  |  |  |  |  |  |  |  |  |  |
| No. Persons | 7 | 4 | 3 |  |  |  |  |  |  |  |  |  |  |  |  |  |  |
| SOC | **27-1011-AG08** | 27-1011 | 27-1013 | 27-1014 | 27-1021 | 27-1024 | 27-1025 | 27-1026 | 27-2012 |  |  |  |  |  |  |  |  |
| Short title | **Designer, artist** | Designer, Art | Artist | Animator | Designer, automotive | Artist, graphic | Designer, Interior | Artists, display | Director, theatre |  |  |  |  |  |  |  |  |
| No. Persons | 23 | 2 | 2 | 1 | 2 | 3 | 4 | 2 | 7 |  |  |  |  |  |  |  |  |
| SOC | **27-2041-AG03** | 27-2041 | 27-2042 | 27-3091 |  |  |  |  |  |  |  |  |  |  |  |  |  |
| Short title | **Musician** | Musician, arranger | Musician, instruments or singers | Interpreter, sign language |  |  |  |  |  |  |  |  |  |  |  |  |  |
| No. Persons | 4 | 1 | 2 | 1 |  |  |  |  |  |  |  |  |  |  |  |  |  |
| SOC | **27-3011-AG04** | 27-3011 | 27-3012 | 27-3022 | 27-3031 |  |  |  |  |  |  |  |  |  |  |  |  |
| Short title | **Press or media** | Announcer, TV | Announcer, other | Reporter, various | Press secretary, PR |  |  |  |  |  |  |  |  |  |  |  |  |
| No. Persons | 11 | 2 | 1 | 4 | 4 |  |  |  |  |  |  |  |  |  |  |  |  |
| SOC | **27-4021-AG04** | 27-4021 | 27-4031 | 27-4032 | 27-4099 |  |  |  |  |  |  |  |  |  |  |  |  |
| Short title | **Theatre, photographer** | Photographer, various | Camera operator | Editor, video | Operator, theatre |  |  |  |  |  |  |  |  |  |  |  |  |
| No. Persons | 5 | 2 | 1 | 1 | 1 |  |  |  |  |  |  |  |  |  |  |  |  |
| SOC | **29-1011-AG06** | 29-1011 | 29-1031 | 29-1041 | 29-1066 | 29-1067 | 29-1069 |  |  |  |  |  |  |  |  |  |  |
| Short title | **Clinician** | Physician, chiropractor | Dietician | Optometrist | Psychiatrist | Surgeon | Cardiologist |  |  |  |  |  |  |  |  |  |  |
| No. Persons | 7 | 1 | 2 | 1 | 1 | 1 | 1 |  |  |  |  |  |  |  |  |  |  |
| SOC | **29-1021-AG04** | 29-1021 | 29-2021 | 31-9091 | 51-9081 |  |  |  |  |  |  |  |  |  |  |  |  |
| Short title | **Dentist, including technicians** | Dentist, general | Dental hygienist | Dental assistant | Technician, dental |  |  |  |  |  |  |  |  |  |  |  |  |
| No. Persons | 27 | 5 | 13 | 8 | 1 |  |  |  |  |  |  |  |  |  |  |  |  |
| SOC | **29-1071-AG06** | 29-1071 | 29-1126 | 29-2054 | 31-1011 | 31-9092 | 31-9097 |  |  |  |  |  |  |  |  |  |  |
| Short title | **Medical assistant** | Medical assistant | Therapist, respiratory | Technician, Respiratory | Aides, home health | Medical assistant | Phlebotomist |  |  |  |  |  |  |  |  |  |  |
| No. Persons | 25 | 3 | 3 | 1 | 10 | 5 | 3 |  |  |  |  |  |  |  |  |  |  |
| SOC | **29-1123-AG07** | 29-1123 | 29-1125 | 29-1127 | 29-1129 | 31-2021 | 31-2022 | 31-9099 |  |  |  |  |  |  |  |  |  |
| Short title | **Therapist** | Therapist, physical | Therapist, recreational | Therapist, speech | Therapist, music | Assistant, physical therapy | Aide, physical therapy | Aide, ortho/prosthetic |  |  |  |  |  |  |  |  |  |
| No. Persons | 16 | 3 | 1 | 3 | 4 | 2 | 2 | 1 |  |  |  |  |  |  |  |  |  |
| SOC | **29-1131-AG02** | 29-1131 | 39-2021 |  |  |  |  |  |  |  |  |  |  |  |  |  |  |
| Short title | **Veteriarian, animal handler** | Veterinarian | Animal shelter workers |  |  |  |  |  |  |  |  |  |  |  |  |  |  |
| No. Persons | 6 | 3 | 3 |  |  |  |  |  |  |  |  |  |  |  |  |  |  |
| SOC | **29-1141-AG05** | 29-1141 | 29-2061 | 31-1013 | 31-1014 | 31-1015 |  |  |  |  |  |  |  |  |  |  |  |
| Short title | **Nurse and patient aides** | Nurse, patient care | Nurse, licensed | Aides, psychiatric | Aides, nursing | Orderlies, hospital |  |  |  |  |  |  |  |  |  |  |  |
| No. Persons | 89 | 60 | 10 | 1 | 17 | 1 |  |  |  |  |  |  |  |  |  |  |  |
| SOC | **29-2011-AG08** | 29-2011 | 29-2012 | 29-2034 | 29-2052 | 29-2053 | 29-2057 | 29-2099 | 29-9099 |  |  |  |  |  |  |  |  |
| Short title | **Technican, laboratory** | Technician, clinical lab | Technician, medical laboratory | Technician, X-Ray | Technician, Pharmacy | Technician, psychiatric | Technician, ophthalmic | Technician, dialysis and others | Technician, medical other |  |  |  |  |  |  |  |  |
| No. Persons | 24 | 3 | 6 | 2 | 1 | 2 | 2 | 4 | 4 |  |  |  |  |  |  |  |  |
| SOC | **33-2011-AG06** | 33-2011 | 33-3021 | 33-3051 | 33-9032 | 33-9092 | 33-9099 |  |  |  |  |  |  |  |  |  |  |
| Short title | **First responder** | Firefighter | Officer, detective | Officer, police | Guard | Lifeguard | Monitor, play ground |  |  |  |  |  |  |  |  |  |  |
| No. Persons | 23 | 2 | 3 | 9 | 4 | 4 | 1 |  |  |  |  |  |  |  |  |  |  |
| SOC | **33-3041-AG03** | 33-3041 | 43-5021 | 43-5052 |  |  |  |  |  |  |  |  |  |  |  |  |  |
| Short title | **Parking officer, bike and postal carriers** | Officer, parking | Messenger, bicycle | Postal carrier |  |  |  |  |  |  |  |  |  |  |  |  |  |
| No. Persons | 6 | 1 | 1 | 4 |  |  |  |  |  |  |  |  |  |  |  |  |  |
| SOC | **35-1011-AG09** | 35-1011 | 35-1012 | 35-2011 | 35-2012 | 35-2015 | 35-2021 | 35-9099 | 51-3011 | 51-3021 |  |  |  |  |  |  |  |
| Short title | **Food preparation/cook** | Chef | Supervisor, food services | Cook, fast food | Cook, institutional | Cook, short order | Food preparation | Food preparation | Baker, bagel | Butcher |  |  |  |  |  |  |  |
| No. Persons | 59 | 2 | 11 | 1 | 20 | 2 | 16 | 1 | 2 | 4 |  |  |  |  |  |  |  |
| SOC | **35-3011-AG05** | 35-3011 | 35-3022 | 35-3031 | 35-3041 | 35-9011 |  |  |  |  |  |  |  |  |  |  |  |
| Short title | **Restaurant/non cook** | Bartender | Attendant, cafeteria | Server, restaurant | Server, non-restaurant | Attendant, bussers |  |  |  |  |  |  |  |  |  |  |  |
| No. Persons | 41 | 3 | 2 | 32 | 1 | 3 |  |  |  |  |  |  |  |  |  |  |  |
| SOC | **35-9021-AG06** | 35-9021 | 37-2011 | 37-2012 | 51-6011 | 51-6031 | 53-7061 |  |  |  |  |  |  |  |  |  |  |
| Short title | **Cleaners and dishwashers** | Cleaner, dishwashers | Custodians, not maids and housekeeping | Housekeeping cleaners | Attendant, laundry | Operator, sewing | Cleaner, various |  |  |  |  |  |  |  |  |  |  |
| No. Persons | 47 | 4 | 21 | 13 | 2 | 4 | 3 |  |  |  |  |  |  |  |  |  |  |
| SOC | **37-1012-AG03** | 37-1012 | 37-3011 | 37-3013 |  |  |  |  |  |  |  |  |  |  |  |  |  |
| Short title | **Grounds keepers** | Supervisor, groundkeeper | Groundkeepers | Tree specialists |  |  |  |  |  |  |  |  |  |  |  |  |  |
| No. Persons | 27 | 1 | 25 | 1 |  |  |  |  |  |  |  |  |  |  |  |  |  |
| SOC | **39-3091-AG02** | 39-3091 | 39-6011 |  |  |  |  |  |  |  |  |  |  |  |  |  |  |
| Short title | **Attendants, bellhops** | Attendants, recreation | Bellhop |  |  |  |  |  |  |  |  |  |  |  |  |  |  |
| No. Persons | 11 | 9 | 2 |  |  |  |  |  |  |  |  |  |  |  |  |  |  |
| SOC | **39-9011-AG03** | 39-9011 | 39-9021 | 39-9032 |  |  |  |  |  |  |  |  |  |  |  |  |  |
| Short title | **Childcare aides** | Childcare providers | Aides, personal care | Aides, childcare, camp |  |  |  |  |  |  |  |  |  |  |  |  |  |
| No. Persons | 18 | 7 | 6 | 5 |  |  |  |  |  |  |  |  |  |  |  |  |  |
| SOC | **41-1011-AG07** | 41-1011 | 41-2011 | 41-2022 | 41-2031 | 41-9011 | 41-9099 | 53-7064 |  |  |  |  |  |  |  |  |  |
| Short title | **Sales, retail** | Manager, retail | Cashiers | Sales, auto and electronic parts | Sales, retail | Sales, demonstrator | Sales, misc | Bagger, retail |  |  |  |  |  |  |  |  |  |
| No. Persons | 113 | 14 | 26 | 3 | 66 | 1 | 1 | 2 |  |  |  |  |  |  |  |  |  |
| SOC | **41-1012-AG09** | 41-1012 | 41-3011 | 41-3021 | 41-3031 | 41-3099 | 41-4011 | 41-4012 | 41-9021 | 41-9022 |  |  |  |  |  |  |  |
| Short title | **Sales, nonretail** | Supervisors, non-retail sales | Account executive, advertising | Sales, insurance | Brokers, financial | Sales, representative | Sales, representative | Broker, commodities | Broker, real-estate | Agents, real estate |  |  |  |  |  |  |  |
| No. Persons | 60 | 2 | 5 | 6 | 2 | 2 | 7 | 21 | 5 | 10 |  |  |  |  |  |  |  |
| SOC | **41-2021-AG07** | 41-2021 | 43-4031 | 43-4061 | 43-4081 | 43-4111 | 43-4131 | 43-4181 |  |  |  |  |  |  |  |  |  |
| Short title | **Clerks, counter, ticker and hotel** | Clerks, car rental | Clerk, legal and motor vehicle | Interviewer | Clerk, hotel registration | Census taker, interviewer | Clear, loan interviewer | Agent, ticket and reservations |  |  |  |  |  |  |  |  |  |
| No. Persons | 15 | 2 | 2 | 2 | 2 | 3 | 2 | 2 |  |  |  |  |  |  |  |  |  |
| SOC | **41-9041-AG03** | 41-9041 | 43-2021 | 43-5031 |  |  |  |  |  |  |  |  |  |  |  |  |  |
| Short title | **Telephone operators** | Sales, telemarketing | Operator, telephone | Operator, telephone |  |  |  |  |  |  |  |  |  |  |  |  |  |
| No. Persons | 8 | 1 | 5 | 2 |  |  |  |  |  |  |  |  |  |  |  |  |  |
| SOC | **43-1011** | 43-1011 |  |  |  |  |  |  |  |  |  |  |  |  |  |  |  |
| Short title | **Clerical, administrative** | Administrative support, clerical |  |  |  |  |  |  |  |  |  |  |  |  |  |  |  |
| No. Persons | 53 | 53 |  |  |  |  |  |  |  |  |  |  |  |  |  |  |  |
| SOC | **43-3021-AG10** | 43-3021 | 43-3031 | 43-3051 | 43-3061 | 43-3071 | 43-4041 | 43-4051 | 43-4071 | 43-4121 | 43-4171 |  |  |  |  |  |  |
| Short title | **Clerk, various** | Clerk, billing | Clerk, financial | Clerk, payroll | Clerk, procurement | Clerk, exchange | Clerk, credit | Clerk, customer service | Clerk, records | Library assistant | Clerk, appointment |  |  |  |  |  |  |
| No. Persons | 71 | 9 | 16 | 4 | 2 | 4 | 4 | 12 | 2 | 2 | 16 |  |  |  |  |  |  |
| SOC | **43-5011-AG07** | 43-5011 | 43-5051 | 43-5061 | 43-5071 | 43-5081 | 43-5111 | 43-9051 |  |  |  |  |  |  |  |  |  |
| Short title | **Agent, mail, cargo, inventory** | Agent, cargo and freight | Clerks, mail room | Clerk, production | Clerk, receiving | Clerk, inventory | Attendants, scale | Clerk, mailroom |  |  |  |  |  |  |  |  |  |
| No. Persons | 42 | 1 | 1 | 6 | 3 | 26 | 4 | 1 |  |  |  |  |  |  |  |  |  |
| SOC | **43-9011-AG02** | 43-9011 | 51-8012 |  |  |  |  |  |  |  |  |  |  |  |  |  |  |
| Short title | **Operator, computer** | Operator, console | Dispatcher, power |  |  |  |  |  |  |  |  |  |  |  |  |  |  |
| No. Persons | 5 | 1 | 4 |  |  |  |  |  |  |  |  |  |  |  |  |  |  |
| SOC | **45-1011-AG03** | 45-1011 | 45-2092 | 47-4031 |  |  |  |  |  |  |  |  |  |  |  |  |  |
| Short title | **Farm worker** | Supervisor, farm workers | Farmworker | Builder, fence |  |  |  |  |  |  |  |  |  |  |  |  |  |
| No. Persons | 13 | 1 | 11 | 1 |  |  |  |  |  |  |  |  |  |  |  |  |  |
| SOC | **45-4021-AG03** | 45-4021 | 47-2061 | 53-1021 |  |  |  |  |  |  |  |  |  |  |  |  |  |
| Short title | **Laborer, construction** | Cutters, wood | Laborer, construction | Supervisor, laborers |  |  |  |  |  |  |  |  |  |  |  |  |  |
| No. Persons | 26 | 1 | 24 | 1 |  |  |  |  |  |  |  |  |  |  |  |  |  |
| SOC | **47-2021-AG04** | 47-2021 | 47-2044 | 47-2051 | 47-3011 |  |  |  |  |  |  |  |  |  |  |  |  |
| Short title | **Trades, mason** | Brick mason | Tile installer | Mason, cement | Helper, mason |  |  |  |  |  |  |  |  |  |  |  |  |
| No. Persons | 5 | 1 | 1 | 1 | 2 |  |  |  |  |  |  |  |  |  |  |  |  |
| SOC | **47-2031** | 47-2031 |  |  |  |  |  |  |  |  |  |  |  |  |  |  |  |
| Short title | **Trades, carpenter** | Carpenter |  |  |  |  |  |  |  |  |  |  |  |  |  |  |  |
| No. Persons | 12 | 12 |  |  |  |  |  |  |  |  |  |  |  |  |  |  |  |
| SOC | **47-2081-AG03** | 47-2081 | 47-2141 | 49-1011 |  |  |  |  |  |  |  |  |  |  |  |  |  |
| Short title | **Trades, painter** | Drywaller | Painter | Supervisor, automotive |  |  |  |  |  |  |  |  |  |  |  |  |  |
| No. Persons | 23 | 4 | 14 | 5 |  |  |  |  |  |  |  |  |  |  |  |  |  |
| SOC | **47-2111-AG03** | 47-2111 | 47-2211 | 49-9021 |  |  |  |  |  |  |  |  |  |  |  |  |  |
| Short title | **Trades, electrical and sheet metal** | Electrician | Sheet metal installer | Installer, HVAC |  |  |  |  |  |  |  |  |  |  |  |  |  |
| No. Persons | 11 | 7 | 1 | 3 |  |  |  |  |  |  |  |  |  |  |  |  |  |
| SOC | **47-2152-AG02** | 47-2152 | 47-5071 |  |  |  |  |  |  |  |  |  |  |  |  |  |  |
| Short title | **Trades, plumbing** | Plumber | Roustabout, oil |  |  |  |  |  |  |  |  |  |  |  |  |  |  |
| No. Persons | 8 | 7 | 1 |  |  |  |  |  |  |  |  |  |  |  |  |  |  |
| SOC | **49-2011-AG05** | 49-2011 | 49-2022 | 49-2094 | 49-9051 | 49-9052 |  |  |  |  |  |  |  |  |  |  |  |
| Short title | **Technican, computer, electrical** | Repair, computer | Telecommunication, repair | Electronics repair | Inspector, electrical | Installer, cable TV |  |  |  |  |  |  |  |  |  |  |  |
| No. Persons | 11 | 3 | 1 | 1 | 3 | 3 |  |  |  |  |  |  |  |  |  |  |  |
| SOC | **49-3011-AG07** | 49-3011 | 49-3021 | 49-3023 | 49-3031 | 49-3091 | 49-9091 | 49-9098 |  |  |  |  |  |  |  |  |  |
| Short title | **Mechanic, vehicle** | Mechanic, aircraft | Autobody repair | Technician, automotive | Mechanic, diesel | Mechanic, bicycle | Mechanic, light | Helper, mechanic |  |  |  |  |  |  |  |  |  |
| No. Persons | 22 | 1 | 3 | 14 | 1 | 1 | 1 | 1 |  |  |  |  |  |  |  |  |  |
| SOC | **49-9012-AG04** | 49-9012 | 49-9041 | 49-9044 | 49-9071 |  |  |  |  |  |  |  |  |  |  |  |  |
| Short title | **Mechanic, industrial** | Mechanic, valve | Mechanic, boiler house | Maintenance, industrial | Maintenance, building |  |  |  |  |  |  |  |  |  |  |  |  |
| No. Persons | 23 | 1 | 1 | 2 | 19 |  |  |  |  |  |  |  |  |  |  |  |  |
| SOC | **51-1011-AG05** | 51-1011 | 51-2092 | 51-2099 | 51-9082 | 51-9199 |  |  |  |  |  |  |  |  |  |  |  |
| Short title | **Production worker** | Supervisor, assembly | Assembly, machine | Assembly, air bag | Technician, medical appliances | Production workers |  |  |  |  |  |  |  |  |  |  |  |
| No. Persons | 103 | 20 | 5 | 3 | 1 | 74 |  |  |  |  |  |  |  |  |  |  |  |
| SOC | **51-4022-AG05** | 51-4022 | 51-4051 | 51-4052 | 51-4072 | 51-4121 |  |  |  |  |  |  |  |  |  |  |  |
| Short title | **Operator, welding and metals** | Operator, forge and machine | Operator, furnace | Foundry worker | Operator, casting | Welder |  |  |  |  |  |  |  |  |  |  |  |
| No. Persons | 18 | 1 | 1 | 2 | 1 | 13 |  |  |  |  |  |  |  |  |  |  |  |
| SOC | **51-4031-AG09** | 51-4031 | 51-4033 | 51-4034 | 51-4035 | 51-4041 | 51-4111 | 51-9032 | 51-9041 | 51-9071 |  |  |  |  |  |  |  |
| Short title | **Operator, machine s** | Operator, press | Operator, tool | Operator, lathe | Operator, milling | Machinist, production | Machinist, tool & die | Operator, cutting machine | Operator, compacting | Operator, stone cutting |  |  |  |  |  |  |  |
| No. Persons | 24 | 1 | 1 | 4 | 2 | 2 | 9 | 2 | 1 | 2 |  |  |  |  |  |  |  |
| SOC | **51-5111-AG03** | 51-5111 | 51-5112 | 51-5113 |  |  |  |  |  |  |  |  |  |  |  |  |  |
| Short title | **Operator, printing** | Technician, photo engraving | Operator, printing | Operator, book binding |  |  |  |  |  |  |  |  |  |  |  |  |  |
| No. Persons | 8 | 3 | 4 | 1 |  |  |  |  |  |  |  |  |  |  |  |  |  |
| SOC | **51-8021-AG05** | 51-8021 | 51-8031 | 51-8091 | 51-8099 | 51-9011 |  |  |  |  |  |  |  |  |  |  |  |
| Short title | **Operator, chemical** | Engineer, boiler and HVAC | Operator, waste | Operator, chemical | Operator, asphalt and cement | Operator, acids |  |  |  |  |  |  |  |  |  |  |  |
| No. Persons | 5 | 1 | 1 | 1 | 1 | 1 |  |  |  |  |  |  |  |  |  |  |  |
| SOC | **53-2011-AG03** | 53-2011 | 53-2012 | 53-2031 |  |  |  |  |  |  |  |  |  |  |  |  |  |
| Short title | **Pilot, flight attendant** | Pilot, commercial | Pilot, civil | Flight attendant |  |  |  |  |  |  |  |  |  |  |  |  |  |
| No. Persons | 4 | 1 | 2 | 1 |  |  |  |  |  |  |  |  |  |  |  |  |  |
| SOC | **53-3021-AG05** | 53-3021 | 53-3022 | 53-3032 | 53-4011 | 53-4031 |  |  |  |  |  |  |  |  |  |  |  |
| Short title | **Driver, heavy duty** | Driver, transit bus | Driver, bus | Driver, truck | Locomotive Engineer | Conductor, freight |  |  |  |  |  |  |  |  |  |  |  |
| No. Persons | 25 | 4 | 2 | 16 | 1 | 2 |  |  |  |  |  |  |  |  |  |  |  |
| SOC | **53-3031-AG04** | 53-3031 | 53-3033 | 53-3041 | 53-6031 |  |  |  |  |  |  |  |  |  |  |  |  |
| Short title | **Driver, light duty** | Driver, delivery | Driver, delivery | Driver, taxi | Attendant, automotive |  |  |  |  |  |  |  |  |  |  |  |  |
| No. Persons | 10 | 2 | 1 | 3 | 4 |  |  |  |  |  |  |  |  |  |  |  |  |
| SOC | **53-7051-AG04** | 53-7051 | 53-7062 | 53-7081 | 53-7199 |  |  |  |  |  |  |  |  |  |  |  |  |
| Short title | **Operator, materials handling** | Operator, forklift | Laborer, materials | Garbage collector | Operator, materials |  |  |  |  |  |  |  |  |  |  |  |  |
| No. Persons | 24 | 1 | 20 | 2 | 1 |  |  |  |  |  |  |  |  |  |  |  |  |
| SOC | **55-3014-AG03** | 55-3014 | 55-3016 | 55-3019 |  |  |  |  |  |  |  |  |  |  |  |  |  |
| Short title | **Military** | Military, artillery | Military, infantry | Military, various |  |  |  |  |  |  |  |  |  |  |  |  |  |
| No. Persons | 19 | 3 | 4 | 12 |  |  |  |  |  |  |  |  |  |  |  |  |  |

## Table S9. Metal Occupational Exposure Score Association with SOC Codes

Single two digit SOC logistic regression models and generalized additive models where the outcome is the metal occupational exposure score, the variables of interest are the number of job-years worked within each two digit SOC code, and the covariates are age, sex, and military service. Interpretation of coefficient is in in terms of five year increments corresponding to standard deviation changes in occupational metal score. LCL, lower confidence limit. UCL, upper confidence limit.

|  |  | **Metal occupational exposure score** | | | |
| --- | --- | --- | --- | --- | --- |
| **Two Digit SOC Code** | **Description** | **Beta** | **95% LCL** | **95% UCL** | **P-Value** |
| 11-0000 | Management Occupations | -0.03 | -0.08 | 0.01 | 0.095 |
| 13-0000 | Business and Financial Operations Occupations | -0.08 | -0.14 | -0.02 | 0.006 |
| 15-0000 | Computer and Mathematical Occupations | -0.07 | -0.15 | 0.01 | 0.083 |
| 17-0000 | Architecture and Engineering Occupations | -0.01 | -0.07 | 0.05 | 0.766 |
| 19-0000 | Life, Physical, and Social Science Occupations | -0.01 | -0.11 | 0.09 | 0.795 |
| 21-0000 | Community and Social Services Occupations | -0.03 | -0.14 | 0.09 | 0.664 |
| 23-0000 | Legal Occupations | -0.07 | -0.17 | 0.03 | 0.189 |
| 25-0000 | Education, Training, and Library Occupations | -0.01 | -0.06 | 0.04 | 0.786 |
| 27-0000 | Arts, Design, Entertainment, Sports, and Media Occupations | -0.03 | -0.11 | 0.04 | 0.361 |
| 29-0000 | Healthcare Practitioners and Technical Occupations | -0.01 | -0.06 | 0.04 | 0.661 |
| 31-0000 | Healthcare Support Occupations | 0.03 | -0.08 | 0.15 | 0.570 |
| 33-0000 | Protective Service Occupations | 0.07 | -0.05 | 0.18 | 0.263 |
| 35-0000 | Food Preparation and Serving Related Occupations | -0.05 | -0.16 | 0.06 | 0.394 |
| 37-0000 | Building and Grounds Cleaning and Maintenance Occupations | 0.12 | 0.02 | 0.22 | 0.016 |
| 39-0000 | Personal Care and Service Occupations | -0.04 | -0.13 | 0.05 | 0.370 |
| 41-0000 | Sales and Related Occupations | -0.08 | -0.14 | -0.02 | 0.011 |
| 43-0000 | Office and Administrative Support Occupations | -0.04 | -0.07 | 0.00 | 0.060 |
| 45-0000 | Farming, Fishing, and Forestry Occupations | 0.01 | -0.13 | 0.15 | 0.867 |
| 47-0000 | Construction and Extraction Occupations | 0.17 | 0.11 | 0.23 | 0.000 |
| 49-0000 | Installation, Maintenance, and Repair Occupations | 0.15 | 0.07 | 0.22 | 0.000 |
| 51-0000 | Production Occupations | 0.18 | 0.14 | 0.22 | 0.000 |
| 53-0000 | Transportation and Material Moving Occupations | -0.04 | -0.12 | 0.04 | 0.359 |
| 55-0000 | Military Occupations | -0.05 | -0.35 | 0.25 | 0.750 |

# Supplemental Figures

## Figure S1. Change in occupational exposure to individual metal subcomponents by time

Each graph shows calendar year (x-axis) by the total percentage of each job that had a self-reported exposure to each of the metal subscores (y-axis) at that time.

### Change in Occupational Exposure to Iron Over Time


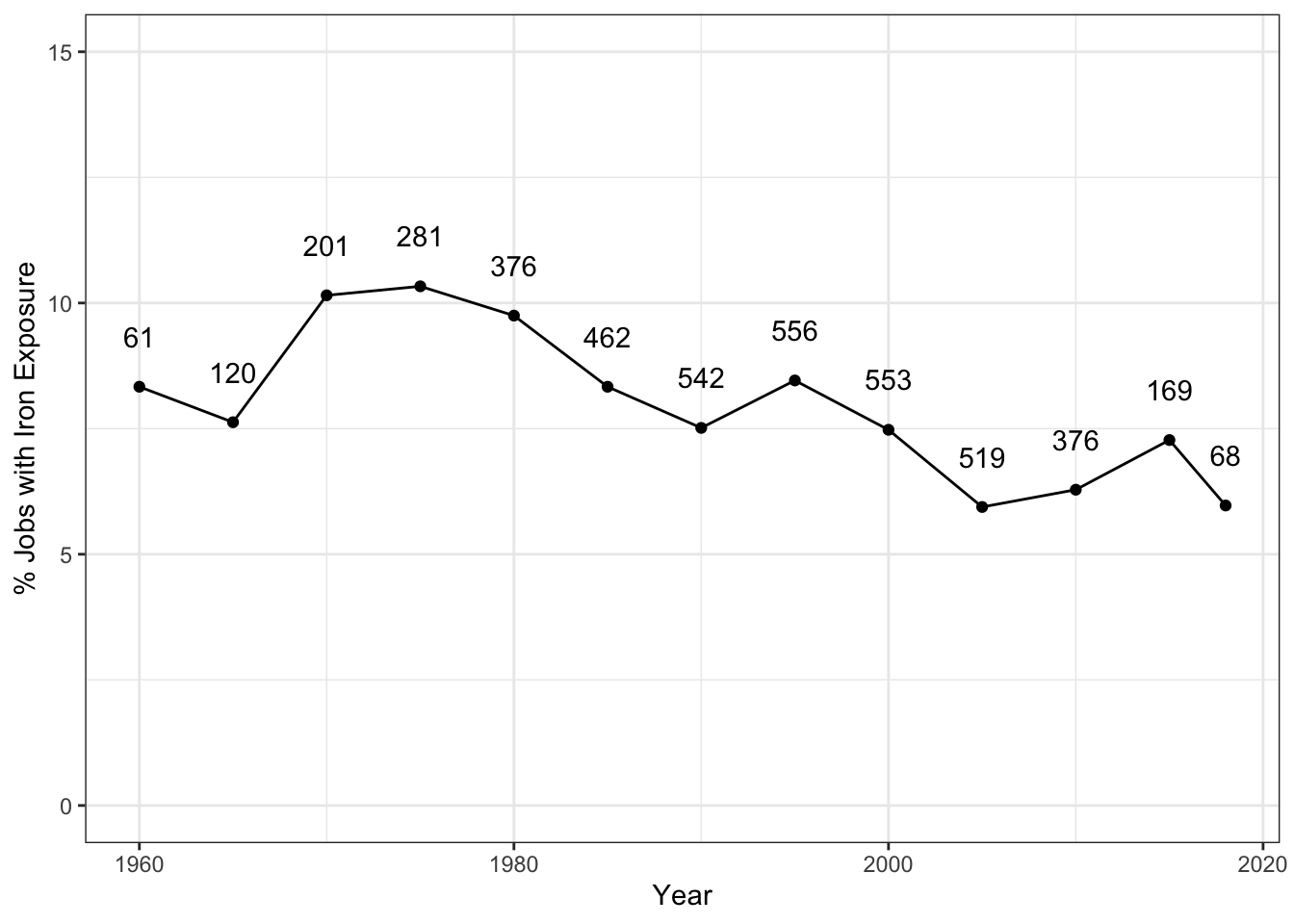


### Change in Occupational Exposure to Lead Over Time


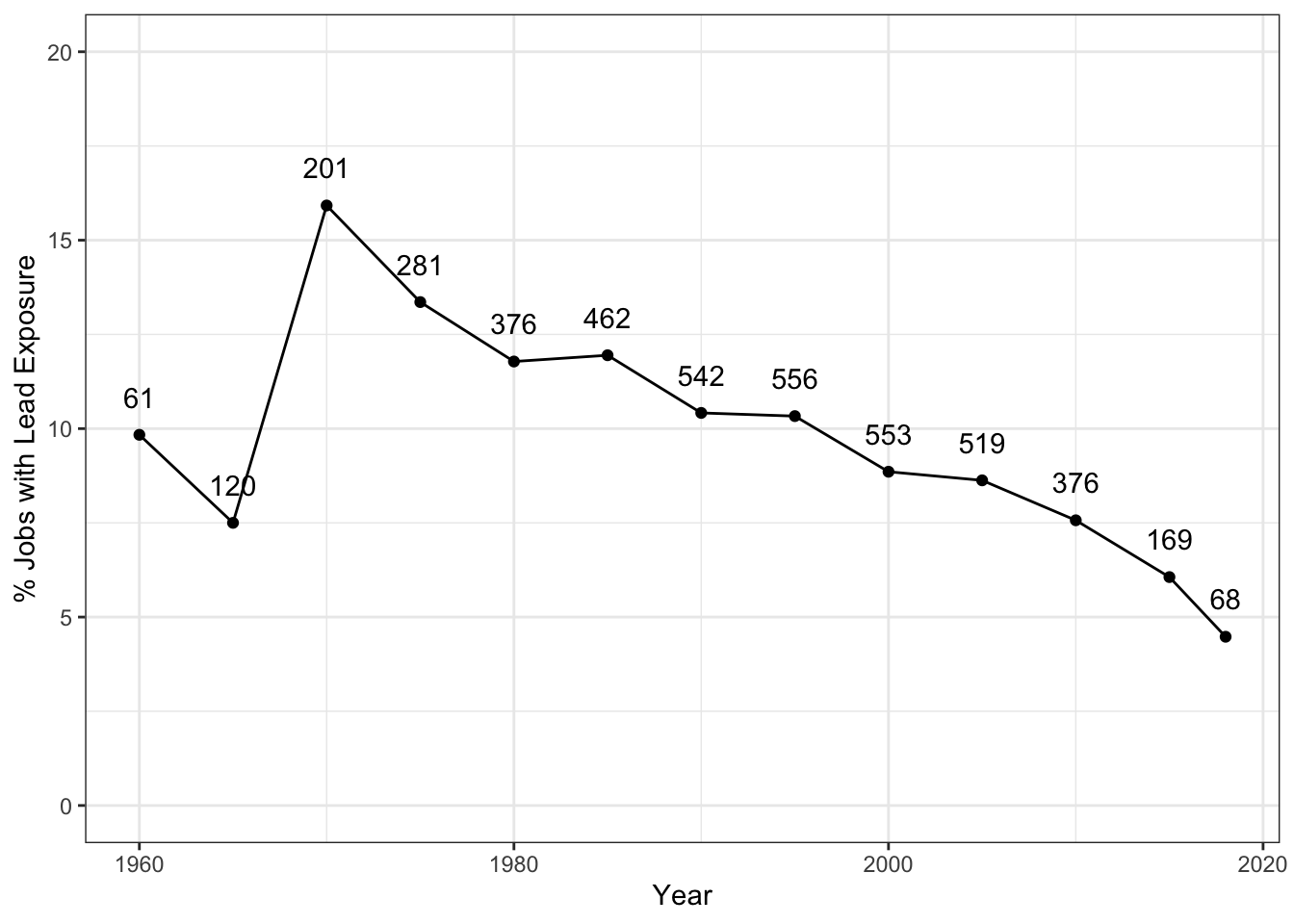


### Change in Occupational Exposure to Mercury Over Time


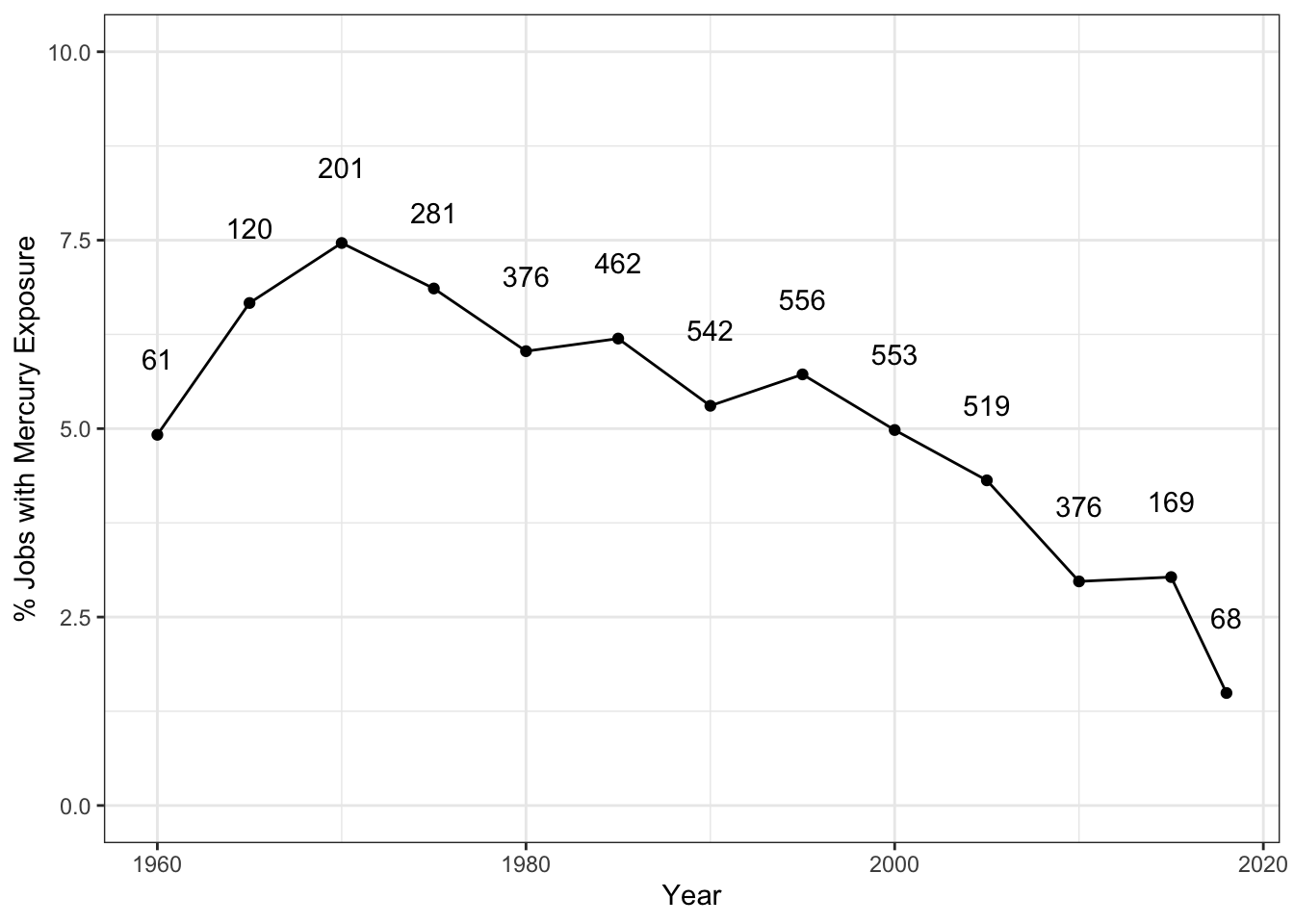


### Change in Occupational Exposure to Cadmium Over Time


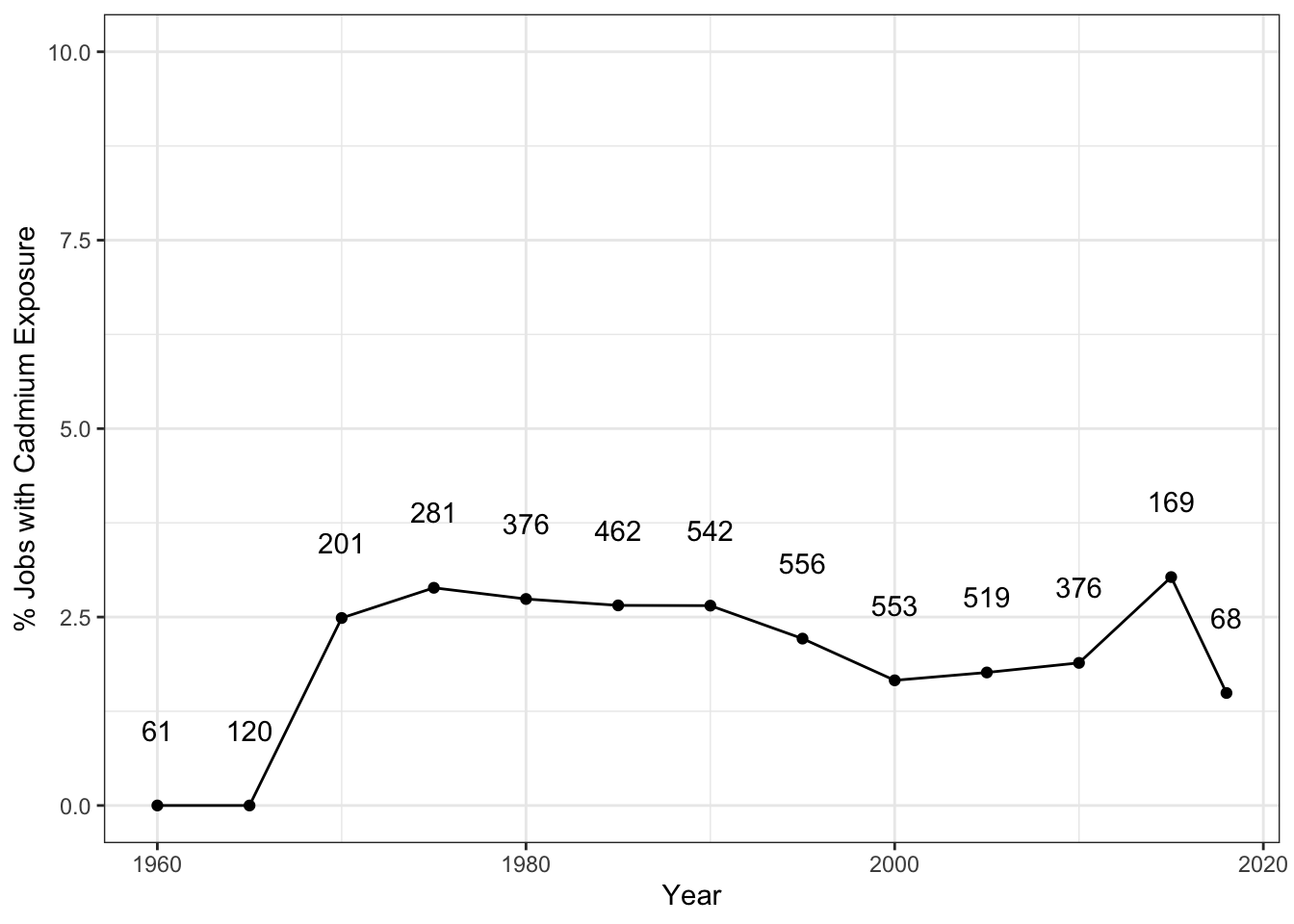


### Change in Occupational Exposure to Beryllium Over Time


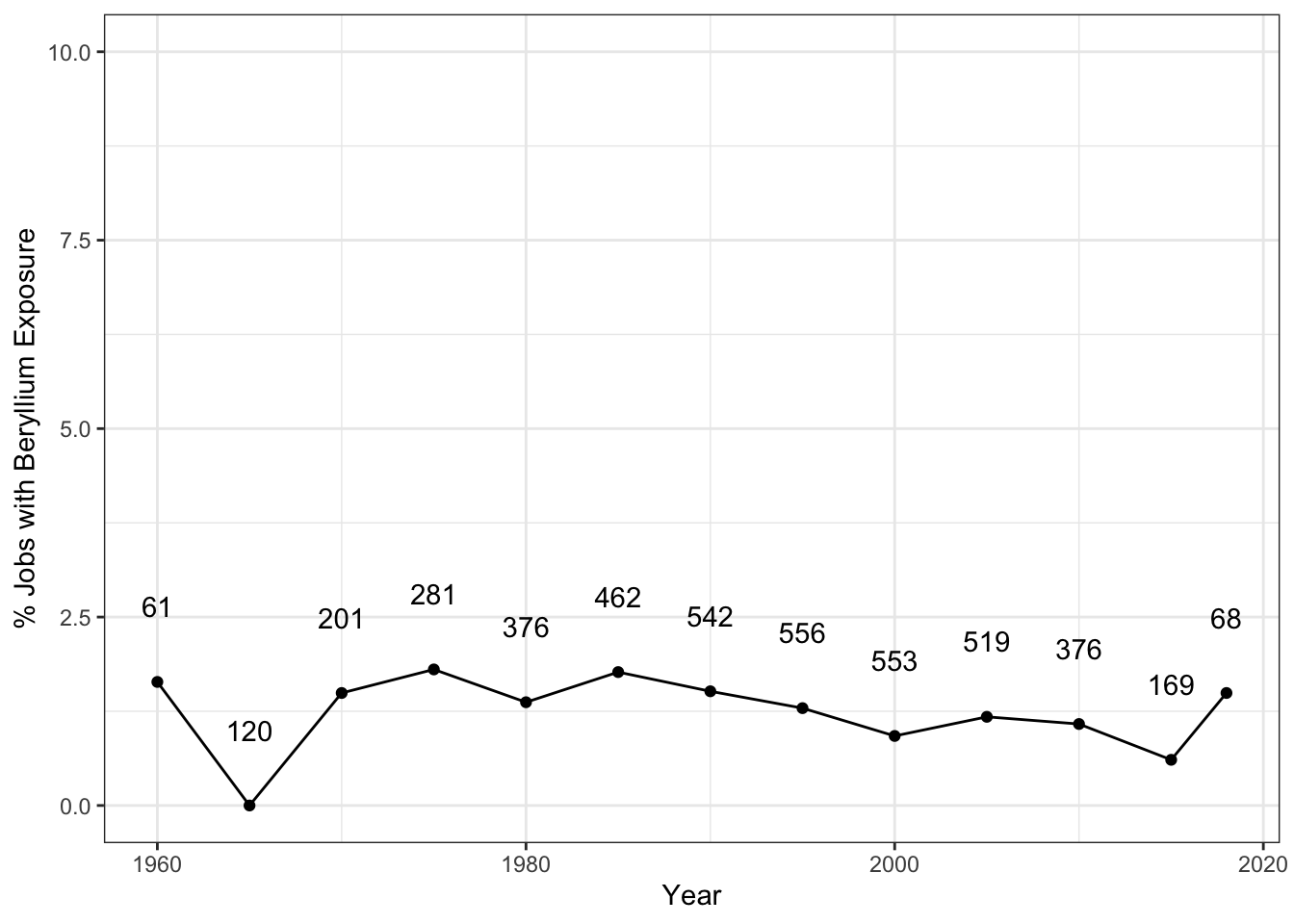


### Change in Occupational Exposure to Nickel Over Time


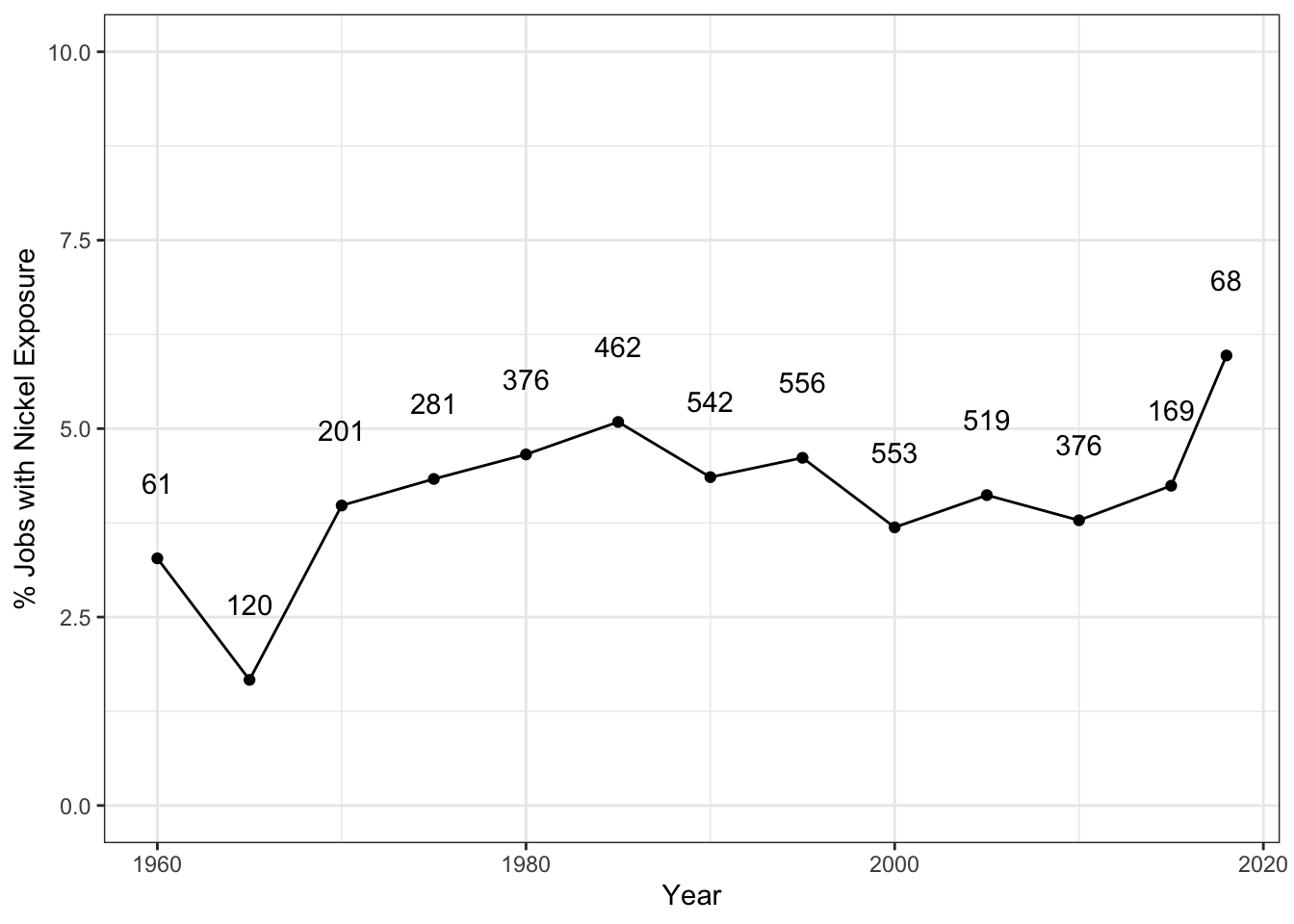


### Change in Occupational Exposure to Aluminum Over Time


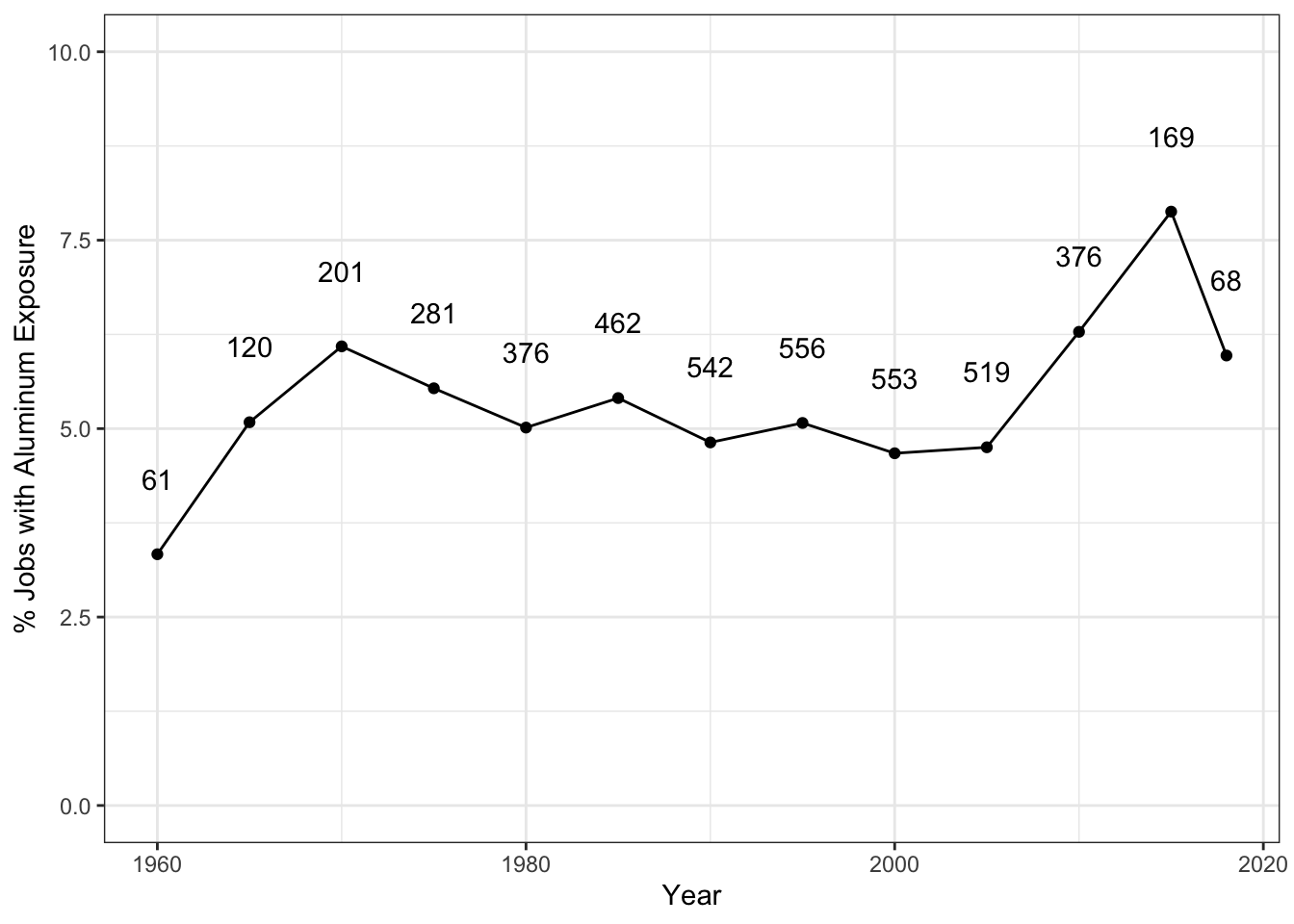


### Change in Occupational Exposure to Arsenic Over Time


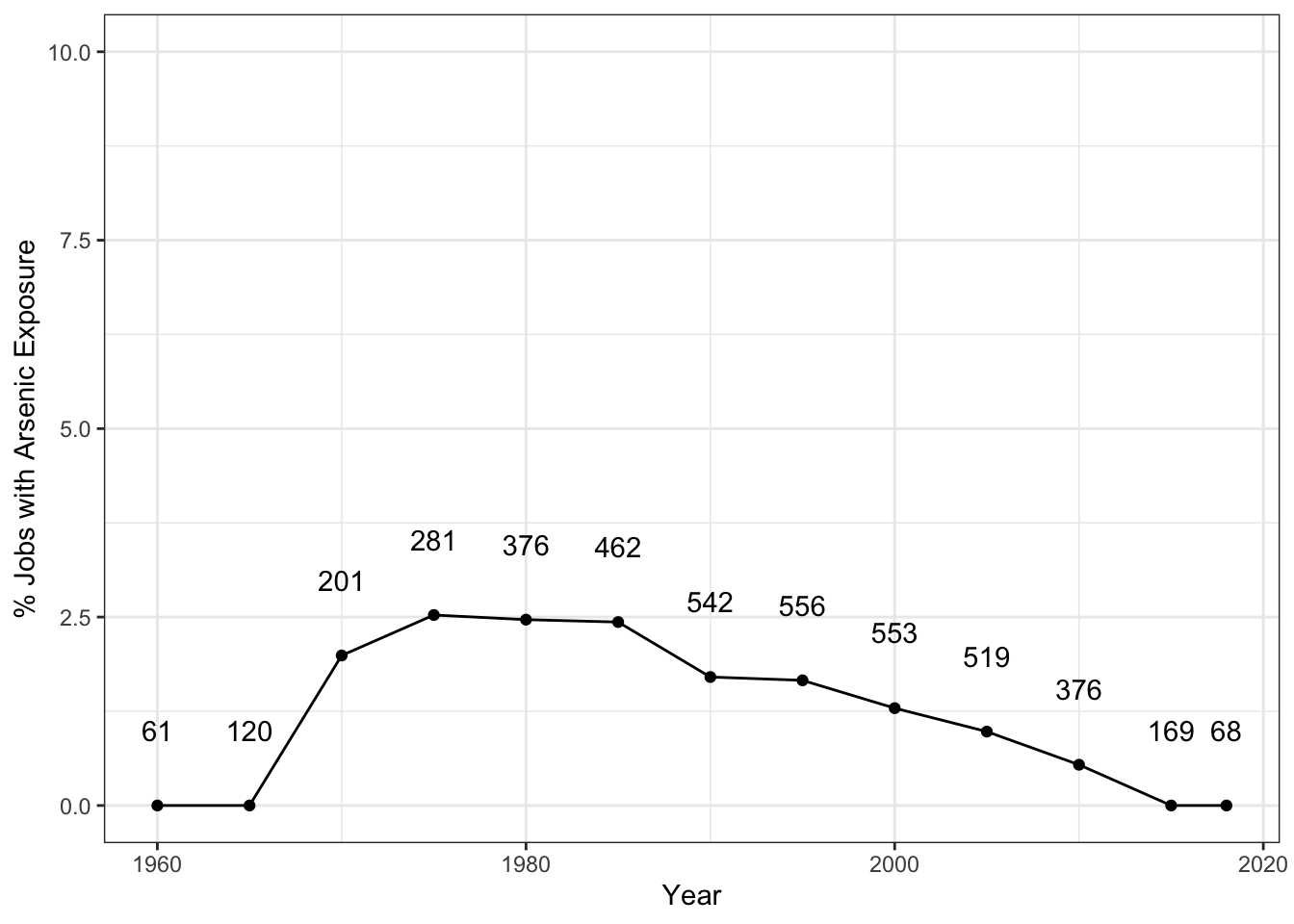


### Change in Occupational Exposure to Welding Fumes Over Time


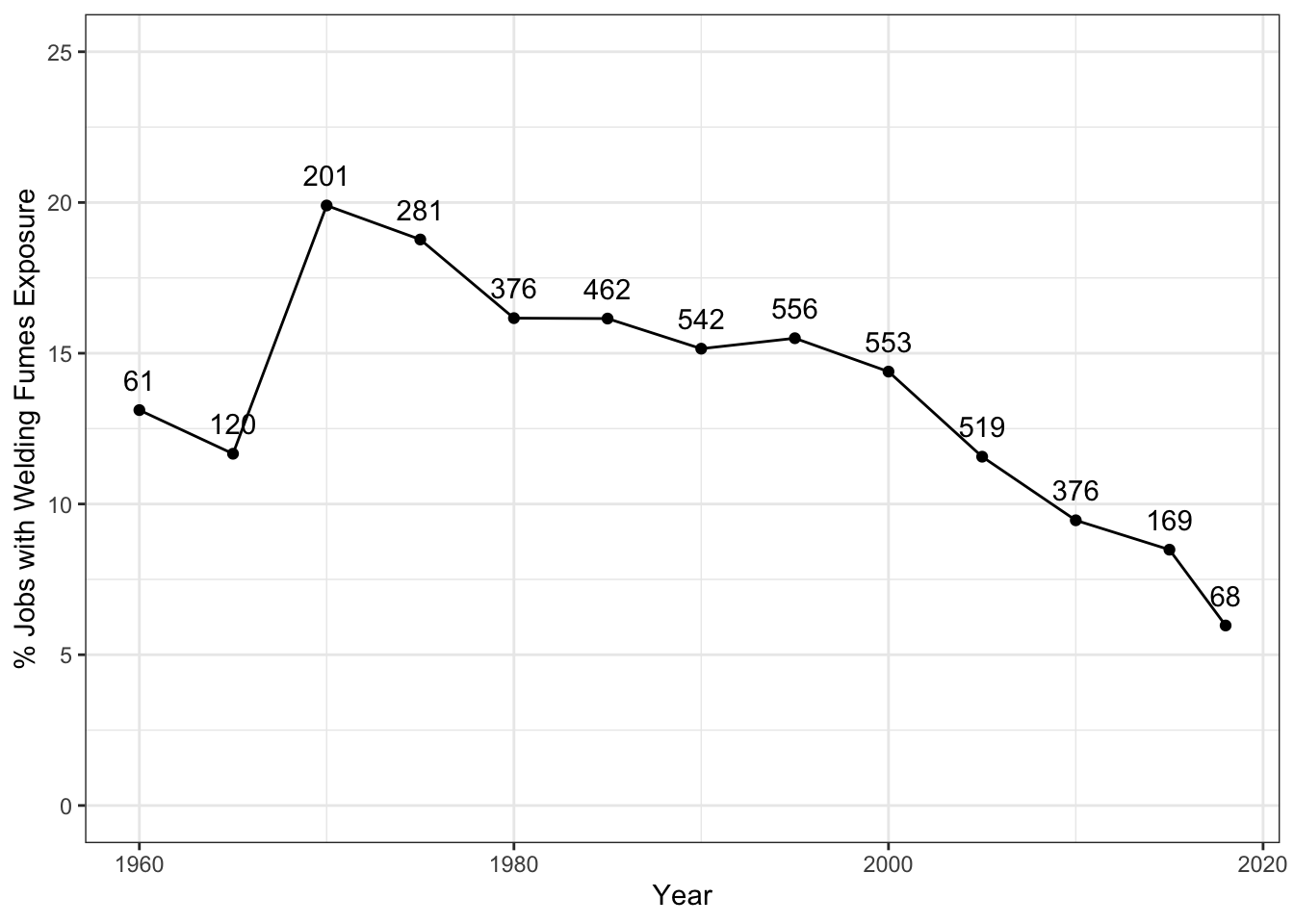

Supplement: Supplementary file 1 — Supplementary file1 (DOCX 959 KB) [file 420_2022_1874_MOESM1_ESM.docx]
